# Anisotropically organized three-dimensional culture platform for reconstruction of a hippocampal neural network

**DOI:** 10.1038/ncomms14346

**Published:** 2017-02-01

**Authors:** So Hyun Kim, Sun-Kyoung Im, Soo-Jin Oh, Sohyeon Jeong, Eui-Sung Yoon, C. Justin Lee, Nakwon Choi, Eun-Mi Hur

**Affiliations:** 1 Center for BioMicrosystems, Brain Science Institute, Korea Institute of Science and Technology (KIST), Seoul 02792, Korea; 2 Center for Neuroscience, Brain Science Institute, Korea Institute of Science and Technology (KIST), Seoul 02792, Korea; 3 Convergence Research Center for Diagnosis, Treatment and Care System of Dementia, Korea Institute of Science and Technology (KIST), Seoul 02792, Korea; 4 Department of Biomedical Engineering, Korea University of Science and Technology (UST), Daejeon 34113, Korea; 5 Department of Neuroscience, Korea University of Science and Technology (UST), Daejeon 34113, Korea; 6 The KU-KIST Graduate School of Converging Science and Technology, Korea University, Seoul 02841, Korea

## Abstract

In native tissues, cellular and acellular components are anisotropically organized and often aligned in specific directions, providing structural and mechanical properties for actuating biological functions. Thus, engineering alignment not only allows for emulation of native tissue structures but might also enable implementation of specific functionalities. However, achieving desired alignment is challenging, especially in three-dimensional constructs. By exploiting the elastomeric property of polydimethylsiloxane and fibrillogenesis kinetics of collagen, here we introduce a simple yet effective method to assemble and align fibrous structures in a multi-modular three-dimensional conglomerate. Applying this method, we have reconstructed the CA3–CA1 hippocampal neural circuit three-dimensionally in a monolithic gel, in which CA3 neurons extend parallel axons to and synapse with CA1 neurons. Furthermore, we show that alignment of the fibrous scaffold facilitates the establishment of functional connectivity. This method can be applied for reconstructing other neural circuits or tissue units where anisotropic organization in a multi-modular structure is desired.

## Abstract (web summary)

Alignment or anisotropic organisation within and between cells enables biological function but is challenging to engineer. Here, the authors align collagen fibres in a pre-strained polydimethylsiloxane mould to generate a 3D scaffold that guides hippocampal neuron axon growth to form CA3–CA1 neural circuits.

Anisotropy is defined as a variation of certain properties of matter when measured along axes in different directions. In native tissues, cellular and extracellular matrix (ECM) components are anisotropically organized and often aligned in specific directions, and here we use the term anisotropy to describe alignment of cells and matrix molecules. In some tissues, alignment is important to provide the tissue with appropriate structural and mechanical properties for actuating unique biological functions: parallel layers of myocardial cells and collagen fibres in the heart provide the structural basis for effective contraction^1^, bundles of aligned fibrillar ECM and tenoblasts in the tendon enable efficient transmission of force^2^, and the highly ordered spatial arrangement of collagen fibrils in corneal stroma is critical for optical transparency^3^. In the developing brain, radial processes of glial cells serve as guidance cables along which new-born neurons migrate from their birthplace to target destinations in the correct lamina of the cerebral cortex. The long radial processes of glia are essential for proper organization of brain^4^,^5^, and dysregulation of such organization has catastrophic consequences to brain function^6^,^7^. Therefore, achieving desired alignment in tissue engineering not only allows emulation of native tissue structure but might also enable implementation of specific functionalities.

In an effort to recapitulate alignment *in vitro*, a number of nano- and microscale technologies have been developed, including topographical patterning^8^,^9^,^10^,^11^, surface chemical modulation^12^, and mechanical deformation^13^,^14^ of culture substrates, but generally for two-dimensional (2D) settings^15^. For three-dimensional (3D) constructs, several strategies have been introduced to confer anisotropy, which in many cases involve application of strong external forces, such as electric^16^,^17^ or magnetic field^18^,^19^ and mechanical stress^19^,^20^,^21^,^22^,^23^. Other approaches utilize cellular tension or traction forces^20^,^24^,^25^,^26^,^27^, which often lead to inhomogeneous alignment. Of note, only a few of such methods that have been devised so far to induce alignment in 3D are compatible with long-term culture of mammalian primary neurons, which are highly sensitive to stress.

Advances in 3D tissue engineering have yielded a growing list of organ- or tissue-mimics, including models of intestine^28^, liver^29^, retina^30^ and the blood-brain barrier^31^. Brain, which is composed of anatomically distinct elements interconnected by neural networks, represents perhaps the most complex organ to reconstruct. The intricate organization of neuronal somata has been modelled by several approaches, such as a multilayered agarose-alginate scaffold that mimics the layered organization of the neocortex^32^, neurospheroid blocks that recapitulate the modular interaction between the cortex and the hippocampus^33^ and a silk-collagen composite scaffold that epitomizes the compartmentalized nature of the brain^34^. Current techniques in stem cell biology are also offering new opportunities to generate brain-mimics^34^,^35^: once a few conditions are met, stem cells assemble spontaneously into neural-tissue clumps that resemble the native laminar structure of the brain. Although aforementioned strategies^32^,^33^,^34^,^35^,^36^ successfully model the modular or layered architecture of neuronal somata, it should be noted that neural connectivity generated within such systems is unorganized because neurite outgrowth occurs randomly in all directions. During development, axons follow precise paths, giving rise to directionally aligned axonal tracts, which is one of the most distinctive anatomical features of the brain. The coherent orientation of axon fibres engenders architectural and mechanical anisotropy of the brain^37^, and alterations in the extent and directionality of anisotropy are associated with a broad range of neurological diseases^38^,^39^,^40^. Despite the importance, little attention has been paid to organizing axon fibres and neural connectivity, especially in 3D constructs.

Here, we introduce a method that applies anisotropic strain during fibrillogenesis of collagen to induce alignment. In particular, this method produces homogeneous alignment of collagen fibrils by altering the shape of a pre-strained polydimethylsiloxane (PDMS) mould containing collagen, before the collagen is fully self-assembled. Applying this method, we have reconstructed the hippocampal CA3–CA1 circuit in a monolithic gel, in which collagen scaffolds aligned in 3D serve as contact guidance cues to direct the growth of axons uniformly across multiple compartments. Furthermore, we have found that functional connectivity between CA3 and CA1 neural populations is markedly promoted by structurally organizing the culture platform.

## Results

### Fabrication of an aligned 3D culture platform

**Figure 1:**
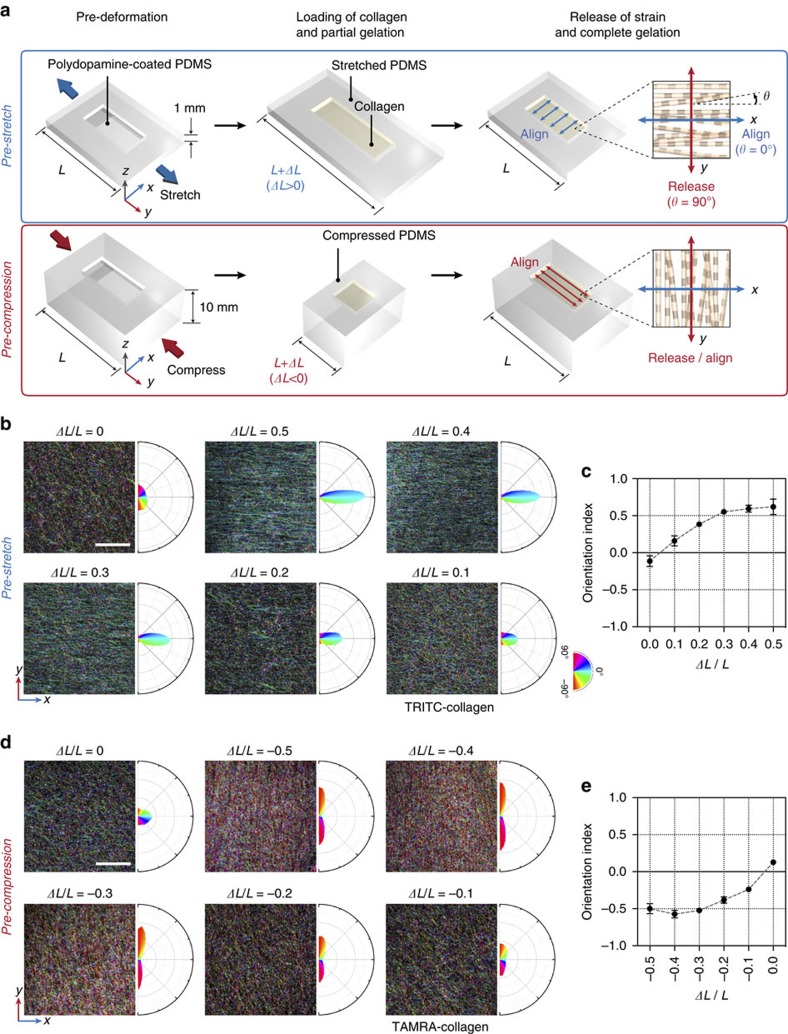
Mechanical pre-deformation of a collagen-containing PDMS chip during fibrillogenesis induces fibril alignment in 3D. (**a**) Schematic illustrating two distinct modes to fabricate fibril-aligned 3D collagen scaffolds. Pre-deformation: PDMS is pre-coated with polydopamine for adhesion of collagen and the PDMS block is either pre-stretched (top) or pre-compressed (bottom) in *y* direction. Loading of collagen and partial gelation: neutralized collagen solution is loaded and allowed to partially gelate in the deformed state. Release of strain and complete gelation: strain is released to restore PDMS to original configuration, in which gelation is completed. Consequently, pre-stretching or pre-compression induces alignment of collagen fibrils in a direction perpendicular (*x* direction) or parallel (*y* direction) to the axis of strain (*y* direction), respectively. *θ* denotes an orientation angle of a collagen fibril with respect to *x* axis. Note that for pre-compression, thicker PDMS chip was designed to prevent the PDMS from being bent or wrinkled. (**b**,**d**) Colour-mapped confocal fluorescence micrographs showing orientation angles of collagen fibrils labelled with TRITC (**b**) or TAMRA (**d**). Polar frequency histograms of orientation angles are presented on right. Strain duration was fixed to 5 min (*t*_D_=5 min), and magnitude of strain (*ΔL*/*L*) was varied from either 0 to 0.5 for pre-stretching (**b**) or 0 to −0.5 for pre-compression (**d**), as indicated. Scale bar, 50 μm. Angular colour scales for the micrographs (left) and the polar frequency histograms (right) are identical, and semicircular colour index for both is presented in the bottom right corner. (**c**,**e**), Plot of mean orientation index calculated from the frequency of orientation angles as a function of *ΔL*/*L*. Error bars in **c**,**e** indicate s.d. from at least three samples.

To fabricate an anisotropically organized 3D culture platform, we devised a simple, yet effective method that induces axial alignment of collagen scaffolds in a PDMS chamber. Exploitation of the elastomeric property of PDMS was essential for alignment, as desired anisotropy was achieved predominantly during restoration of a pre-deformed PDMS channel to its original configuration. As illustrated in Fig. 1a, the PDMS channel was pre-deformed (either stretched or compressed) by applying uniaxial strain along the *y* axis to a certain extent (from *L* to *L*+Δ*L*). Then, a collagen solution was loaded into the pre-deformed channel and fibrillogenesis was initiated. After briefly holding the PDMS chip in the deformed state, strain was released while the collagen was still in the sol/gel biphasic state, and PDMS returned to original configuration. Restoration induced transformation of the viscoelastic collagen matrix confined in the PDMS chip, accordingly. To achieve desired alignment, it was crucial to release strain before the loaded collagen was fully self-assembled, so that fibrillogenesis was completed in the restored configuration.

### Optimizing conditions for alignment

We varied the magnitude and the duration of strain to optimize conditions for alignment. Throughout the text, magnitude of strain is presented as a ratio of deformed versus original length (*ΔL*/*L*), and positive and negative values denote pre-stretch and pre-compression, respectively. Strain duration is depicted as *t*_D_, which indicates how long the PDMS chip was held in the deformed configuration. Collagen was labelled with either tetramethylrhodamine isothiocyanate (TRITC) or 5-carboxytetramethylrhodamine (TAMRA) to visualize positions and angular distributions of fibrils. From fluorescence micrographs of the TRITC- or TAMRA-labelled collagen fibrils, we calculated orientation indices (OIs), defined as 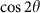, where *θ* denoted the local orientation angle of a collagen fibril relative to the *x* axis (see ‘Methods' section and Fig. 1a). According to this definition, *θ* values ranging from −90° to 90° produce OIs between 1 and −1. OI values of 1 and −1 represent perfect alignments orthogonal and parallel to the axis of strain, respectively, and OI value of 0 represents a completely isotropic condition.

To optimize the magnitude of strain, we varied *ΔL*/*L* from −0.5 to 0.5 while holding constant *t*_D_ for 5 min, and calculated the OI values from fluorescence micrographs. In both pre-stretched (Fig. 1b,c) and pre-compressed (Fig. 1d,e) conditions, greater deformation produced better alignment up to 30%, and then reached a plateau. Notably, pre-stretching and pre-compression along the same axis (*y* axis) produced alignments in perpendicular directions: pre-stretching induced alignment along the *x* axis, whereas pre-compression induced alignment along the *y* axis.

**Figure 2:**
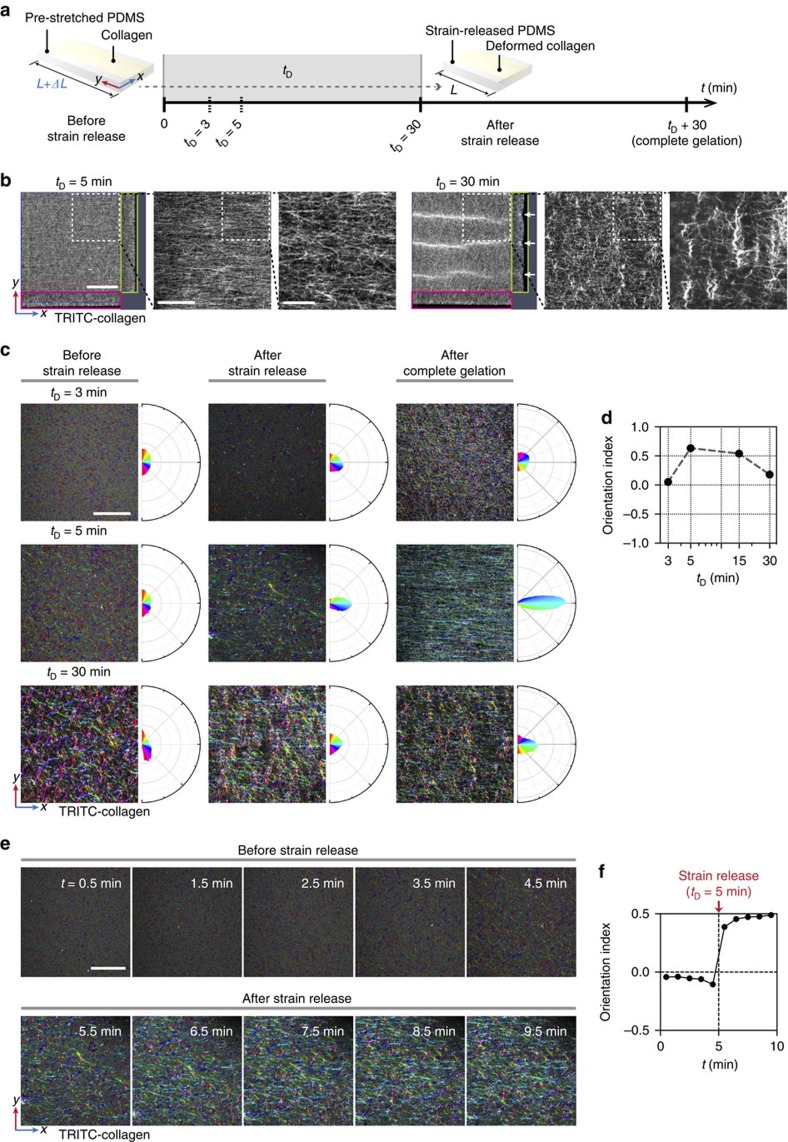
Optimization of strain duration for desired alignment of collagen fibrils. (**a**) Schematic illustrating configurations of collagen-containing PDMS chips over time during the course of mechanical pre-deformation and restoration. *t*_D_ values indicate the time when strain was released. As a first step, a PDMS chip is pre-stretched by *ΔL* and collagen solution is loaded. At a certain *t*_D_, strain is released and PDMS chip returns to original configuration (*L*), causing collagen to be deformed accordingly. Gelation is completed for additional 30 min after *t*_D_. (**b**) Confocal fluorescence micrographs of collagen gels. Pre-deformed PDMS chips (*ΔL*/*L*=0.5) were loaded with TRITC-labelled collagen, held in the deformed state for either *t*_D_=5 min (left) or *t*_D_=30 min (right), and then the strain was released. Confocal fluorescence micrographs were acquired after gelation was completed. Left panels, composite images (*xy* planes) and orthogonal views (*xz* and *yz* planes) from *z* stacked images (stack size, 50 μm; step size, 1 μm). Scale bar, 100 μm. Middle and right panels, images of single focal planes. Scale bars, 50 μm in middle, 20 μm in right. White arrows indicate furrows on the buckled surface for *t*_D_=30 min. (**c**) Colour-mapped confocal fluorescence micrographs of TRITC-labelled-collagen fibrils right before strain release (left panels), immediately after strain release (middle panels), and after complete gelation (right panels), for *t*_D_ of 3, 5 and 30 min. Polar frequency histograms of orientation angles are shown on right of each panel. Angular colour scales for micrographs and polar frequency histograms are identical to Fig. 1b. Scale bar, 50 μm. (**d**) Log-linear plot of OI calculated from the frequency of orientation angles presented in the right panels of (**c**). (**e**) Temporal imaging of TRITC-labelled-collagen fibrils before (top panels) and after strain release (bottom panels), for *t*_D_=5 min. Angular colour scale is identical to Fig. 1b. Scale bar, 50 μm. (**f**) Plot of OI calculated from the frequency of orientation angles presented in **e**. Red arrow indicates *t*_D_ (5 min).

We then optimized *t*_D_ by pre-stretching PDMS chips to a fixed ratio (*ΔL*/*L*=0.5) and holding the collagen-loaded chips in the deformed state for various durations (Fig. 2a). We found that when *t*_D_ was 5 min, collagen fibrils aligned homogeneously throughout the gel (Fig. 2b). When *t*_D_ was 30 min, wrinkle formation was evident on the surface of the gelated collagen block and buckled fibrils were observed throughout the bulk (Fig. 2b). Although wrinkled surface topology could have widespread applications^41^, here we aimed at finding conditions that produce homogeneous alignment throughout the gel.

The mechanisms of collagen fibrillogenesis are still not fully understood, but the process has been consistent with the nucleation and subsequent growth model of collagen self-assembly^42^. While soluble collagens (collagen monomers) self-assemble into aggregates (that is, nucleates) and form fibrils, the process of fibrillogenesis has been shown to fit into a sigmoidal curve with a distinct lag phase before the onset of a growth phase, which is followed by a plateau phase. The lag phase is interpreted as the period of aggregate formation, and the growth phase as the period of linear and lateral addition of collagen. According to this self-assembly model, fibrillogenesis is initiated in the pre-deformed PDMS chamber, and soluble collagens start to assemble into fibrils of various lengths. Releasing strain along the *y* axis after a certain *t*_D_ induces compression of the collagen matrix in *y* dimension but extension in *x* and *z* dimensions. In a micrometre scale, this step reorients the randomly oriented short fibrils surrounded by the viscoelastic matrix, causing fibrils to align along the transverse direction of net compression (that is, align parallel to the direction of net stretch). If *t*_D_ is short (for example, 3 min), soluble collagens represent a major portion, and thus in the restored PDMS chip, soluble collagen molecules assemble into fibrils without consistent directionality. If *t*_D_ gets longer (for example, 5–15 min), the portion of short fibrils reaching a certain length (which can be detected under a fluorescence microscope) increases at the expense of soluble collagens. When the chip and the matrix transform shape in response to strain release, such short fibrils change orientation and become aligned. In the restored chip, aligned fibrils serve as templates for further fibrillogenesis, producing anisotropically organized fibre network. Indeed, TRITC-labelled collagen fibrils were readily detected at 5 min but not at 3 min (Fig. 2c,d). Moreover, we confirmed that fibril orientation changed abruptly by releasing strain at 5 min (Fig. 2e,f and Supplementary Movie 1). If *t*_D_ gets even longer (for example, 30 min) and collagen fibrils exceed certain length, strain release no longer effectively orients collagen fibrils, probably because of restricted space in the dense fibre network. We confirmed that when *t*_D_=30 min, long fibrils were formed (Fig. 2c). Therefore, releasing strain within a certain range of *t*_D_ is essential for alignment (Fig. 2d).

### Analytical models of collagen fibril alignment in 3D

To provide an explanation for alignment, we propose analytical models illustrated in Supplementary Figs 1 and 2. Because of the nonlinear elastic property of PDMS, when a PDMS chip is pre-deformed by *λ*, defined as 1+*ΔL*/*L*, in an axial (*y*) direction, it is also deformed in two transverse (*x* and *z*) directions by *λ*^−1/2^. Similarly, as the pre-deformed PDMS chip returns to original configuration by releasing the axial strain after *t*_D_, the viscoelastic collagen matrix confined in the PDMS chip is deformed by *λ*^−1^ in the axial (*y*) direction and *λ*^1/2^ in the two other transverse (*x* and *z*) directions. Deformations in both axial and transverse directions synergistically induce alignment; the axial deformation by *λ*^−1^ serves as a dominant driving force to align fibrils while the transverse deformation by *λ*^1/2^ contributes additionally. Supposing that a collagen fibril is positioned in a certain volume of a viscoelastic matrix and that the matrix volume is identical before and after strain release, fibril orientation after strain release can be expressed as 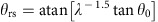 in the *xy* plane, 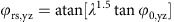 in the *yz* plane, and 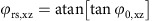 in the *xz* plane, where *θ* and *ϕ* are azimuthal and polar angles of the fibril, respectively, and subscripts 0 and rs denote pre-deformed and restored conditions, respectively. Specifically, when strain is released from the pre-stretched PDMS chip in *y* direction, the matrix containing the fibril is consequently compressed in *y* direction while being stretched in both *x* and *z* directions. Both compression in *y* direction and stretch in *x* direction contribute the alignment of the fibril along *x* direction; the azimuthal angle of the collagen fibril decreases (*θ*_rs_<*θ*_0_; Supplementary Fig. 1). Of note, stretching in *z* direction has no effect on the alignment along *x* direction because the polar angle remains identical in the *xz* plane (*ϕ*_rs,xz_=*ϕ*_0,xz_) and changes in the polar angle in the *yz* plane are orthogonally independent of desired alignment along *x* direction. Conversely, as the pre-compressed PDMS chip returns to original configuration, extension in *y* direction and compression in *x* direction induce alignment of the fibril along *y* direction; the azimuthal angle of the collagen fibril increases (*θ*_rs_>*θ*_0_; Supplementary Fig. 2). Compression in *z* direction supports the alignment along *y* direction because the polar angle in the *yz* plane decreases (*ϕ*_rs,yz_<*ϕ*_0,yz_).

### Reconstruction of a hippocampal 3D neural network

**Figure 3:**
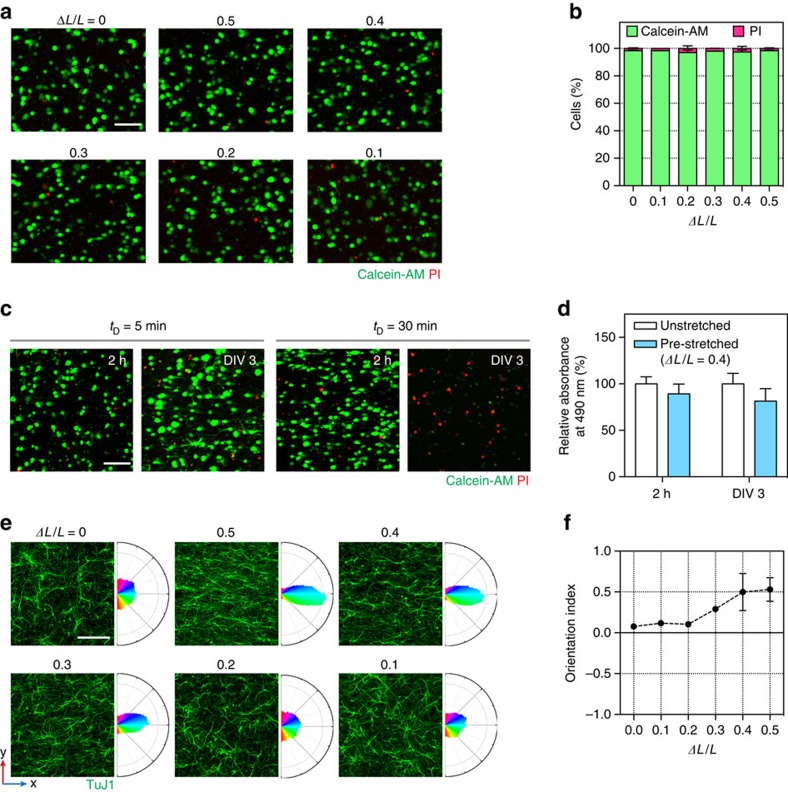
Effect of strain magnitude and duration on neuronal cell viability and alignment. (**a**,**b**) Shown are representative confocal fluorescence micrographs (maximum intensity projection images) of live and dead embryonic (E18.5) hippocampal neurons stained with calcein-AM and PI, respectively (**a**) and quantification of cell viability (**b**). Cell-seeded collagen solutions were loaded into single-channel PDMS chips that were pre-stretched to varying ratios (*ΔL*/*L*=0 to 0.5), as indicated. After 5 min, strain was released and gelation was completed. Cell viability was assessed after 2 h. (**c**) Confocal fluorescence micrographs of embryonic (E18.5) hippocampal neurons stained with calcein-AM and PI. Pre-deformed PDMS chips (*ΔL*/*L*=0.5) were loaded with a cell-laden collagen solution, held in the deformed state either for 5 or 30 min, as indicated, and then the strain was released. Cells viability was assessed at 2 h (left) or 3 days (DIV 3; right) after completion of gelation, as indicated. Scale bar, 100 μm. (**d**) MTS assay was performed with neurons growing in pre-stretched (*ΔL*/*L*=0.4) or unstretched (*ΔL*/*L*=0) PDMS chips at 2 h or DIV 3 after gelation. Error bars indicate s.d. from at least three samples. (**e**) Confocal fluorescence micrographs (maximum intensity projection images) showing neurites of embryonic (E18.5) hippocampal neurons stained with TuJ1 (left) and polar frequency histograms of orientation angles of the neurites (right). Magnitude of strain (*ΔL*/*L*) was varied from 0 to 0.5, as indicated, while holding *t*_D_ constant for 5 min. Angular colour scales for polar frequency histograms are identical to Fig. 1b. Scale bar, 200 μm. (**f**) Plot of orientation index calculated from orientation angles of neurites. Error bars indicate s.d. from at least three samples.

Next, we extended this approach to generate a contact guidance field between different populations of neurons and induce directionally aligned axon growth from one population of neurons to another. As mammalian primary neurons are notoriously sensitive to shear stress and mechanical distortion, we first examined if neuronal cell viability was affected by the pre-deformation and restoration procedure. Live/dead cell viability assay with calcein-AM and propidium iodide (PI) revealed that cell viability was unaffected by varying the extent of deformation up to *ΔL*/*L*=0.5, and most neurons (98±0.61%) survived the procedure (Fig. 3a,b) when *t*_D_ was set at 5 min. In contrast to *ΔL*/*L*, strain duration had a profound effect on cell viability. Although most neurons were alive for a couple of hours regardless of *t*_D_, few neurons survived 3 days or longer when *t*_D_ was 30 min (Fig. 3c). Therefore, releasing strain within a certain *t*_D_ was vital for survival. Consistent with the calcein-AM/PI doubling staining, MTS cytotoxicity assay confirmed that the pre-deformation and restoration procedure had little cytotoxicity when *ΔL*/*L* was 0.4 and *t*_D_ was 5 min (Fig. 3d). We found that neurons underwent differentiation regardless of the extent of strain, as long as *t*_D_ was 5 min (Fig. 3e,f). Of note, axons in the pre-stretched PDMS chip aligned in *x* axis and calculation of OI values confirmed that mechanical deformation to a greater extent produced better alignment of axons, followed by a plateau (Fig. 3e,f).

**Figure 4:**
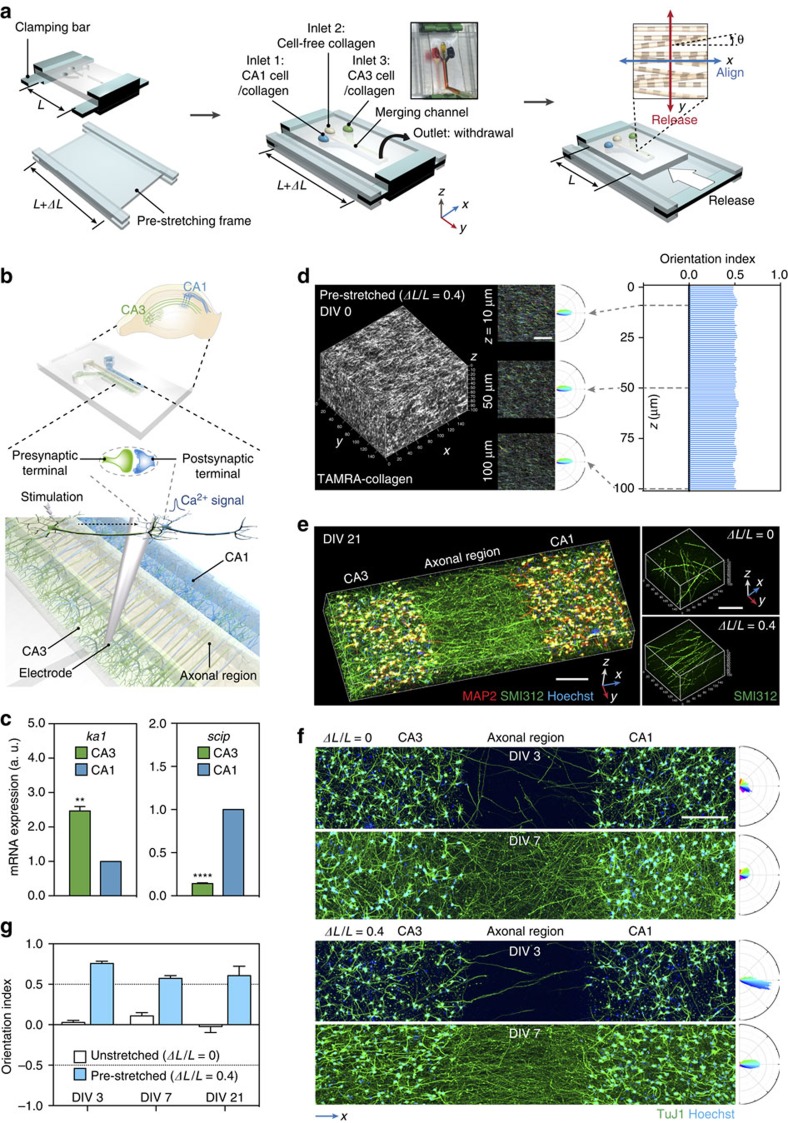
Formation of an anisotropically organized CA3–CA1 neural circuit. (**a**) Schematic for reconstruction of an anisotropically organized, hippocampal neural network. A 3-channel PDMS chip was pre-stretched (from *L* to *L*+*ΔL*) and inlets were loaded with CA1 cell-seeded, cell-free and CA3 cell-seeded collagen, as indicated. Alignment was induced as illustrated in Supplementary Fig. 4. (**b**) Schematic of the reconstructed CA3–CA1 network. Note that axons running parallel to *x* axis grow across the axonal compartment and connect the two (CA3 and CA1) somatodendritic compartments. A synapse formed between a CA3 (presynaptic) and a CA1 (postsynaptic) neuron is illustrated. (**c**) Levels of *ka1* and *scip* mRNA isolated from rat hippocampal areas CA3 and CA1 (normalized to *gapdh*). Values are presented in a.u. Error bars indicate s.e. of mean (*n*=3; s.d. of 0.2212 and 0.01458 for *ka1* and *scip1* from CA3, respectively). **and ****P=7.50 × 10^−3^ and 9.64 × 10^−5^, respectively (parametric unpaired *t*-tests with Welch's correction assuming unequal s.d.). (**d**) Confocal fluorescence micrograph of TAMRA-labelled collagen fibrils rendered in 3D (160 μm × 160 μm × 100 μm) in a pre-stretched (*ΔL*/*L*=0.4), cell-seeded PDMS chip. Insets, colour-mapped confocal micrographs of collagen fibrils at *z=*10, 50, and 100 μm, as indicated. Next to micrographs are polar frequency histograms of orientation angles. Scale bar, 50 μm. Graph on right shows orientation index of collagen fibrils throughout depth of 100 μm. (**e**) Left, 3D rendering (1919.27 μm × 638.92 μm × 100 μm) of the CA3–CA1 culture (DIV 21) immunostained for MAP2 (dendrites), SMI312 (axons) and Hoechst. Each channel is visualized in Supplementary Movie 3. Scale bar, 200 μm. Right, 3D rendering of a region in axonal compartment (160 μm × 160 μm × 100 μm) in unstretched (*ΔL*/*L*=0) and pre-stretched (*ΔL*/*L*=0.4) PDMS chips (axons in Supplementary Movies 4 and 7; collagen fibrils in Supplementary Movies 5 and 6). Scale bar, 100 μm. (**f**) Confocal fluorescence micrographs of CA3–CA1 cultures in unstretched (*ΔL*/*L*=0) and pre-stretched (*ΔL*/*L*=0.4) PDMS chips. Scale bar, 200 μm. Cultures were immunostained for neurites (TuJ1) and Hoechst at DIV 3 and 7. Polar frequency histograms of orientation angles of neurites (axonal region) are presented on right. Angular colour scales in **d**,**e** are identical to Fig. 1b. (**g**) Orientation indices of neurites (axonal compartment) of unstretched and pre-stretched PDMS chips at DIV 3, 7 and 21, as indicated. Error bars indicate s.d.

We applied this method to reconstruct the hippocampal CA3–CA1 circuit, which represents perhaps one of the most intensively studied neural circuits for investigating the principles that underlie neural connectivity and synaptic plasticity. For this purpose, we designed a PDMS chip with three inlets that merge into a main channel with a common outlet (Fig. 4a). The chip was pre-stretched along *y* axis and then released to create aligned matrix (collagen fibrils) perpendicular to the direction of strain, that is, across the three compartments. In that way, axons would be guided by the aligned matrix and connect the two somatodendritic (CA3 and CA1) compartments located apart from each other (Fig. 4b). Areas CA3 and CA1 from rat embryonic hippocampi were dissected out and isolation of the target regions was confirmed by performing quantitative real-time-polymerize chain reaction (qRT-PCR) from the tissues using primers for region-specific genes, *scip* (*suppressed cAMP-inducible POU*) and *ka1* (*kainite receptor*; *GRIK4*)^43^ (Fig. 4c). On the basis of the results described above and additional confirmation of alignment by scanning electron microscopy (Supplementary Fig. 3), *ΔL*/*L* was set to 0.4 and *t*_D_ to 5 min. Cell-free or cell-embedded collagen solutions were loaded into the three inlet reservoirs (Fig. 4a), and by withdrawal from a common outlet, the merging channel was filled by laminar co-flows of collagen solutions. Adjacent streams of collagen remained separate in millimeter-scale, which provided a framework for the construction of compartmentalized regions. Immediately after the completion of gelation, the 3D culture chip was placed in a Petri dish containing sufficient volume of media (see ‘Methods' section and Supplementary Fig. 4).

In pre-stretched chips (*ΔL*/*L*=0.4), homogeneous alignment of collagen fibrils was evident throughout the gel (Fig. 4d and Supplementary Movie 2), and we also confirmed directional axon growth, especially in the axonal compartment (Fig. 4e,f, Supplementary Fig. 5 and Supplementary Movies 3–5). OI values from days *in vitro* (DIV) 21 chips were slightly smaller than those from DIV 0 (Supplementary Fig. 6), presumably because of degradation of the hydrogel by cells^44^,^45^. Nevertheless, alignment of both the collagen fibrils and axons was evident throughout the entire gel at DIV 21. By contrast, in unstretched chips (*ΔL*/*L*=0), orientations of fibrils (Supplementary Movie 6) and axon growth did not show any consistent directionality (Fig. 4e,f, Supplementary Fig. 5 and Supplementary Movie 7). These results suggest that directionally aligned collagen fibrils serve as topological cues for axon growth and guide the otherwise randomly growing axons in 3D.

**Figure 5:**
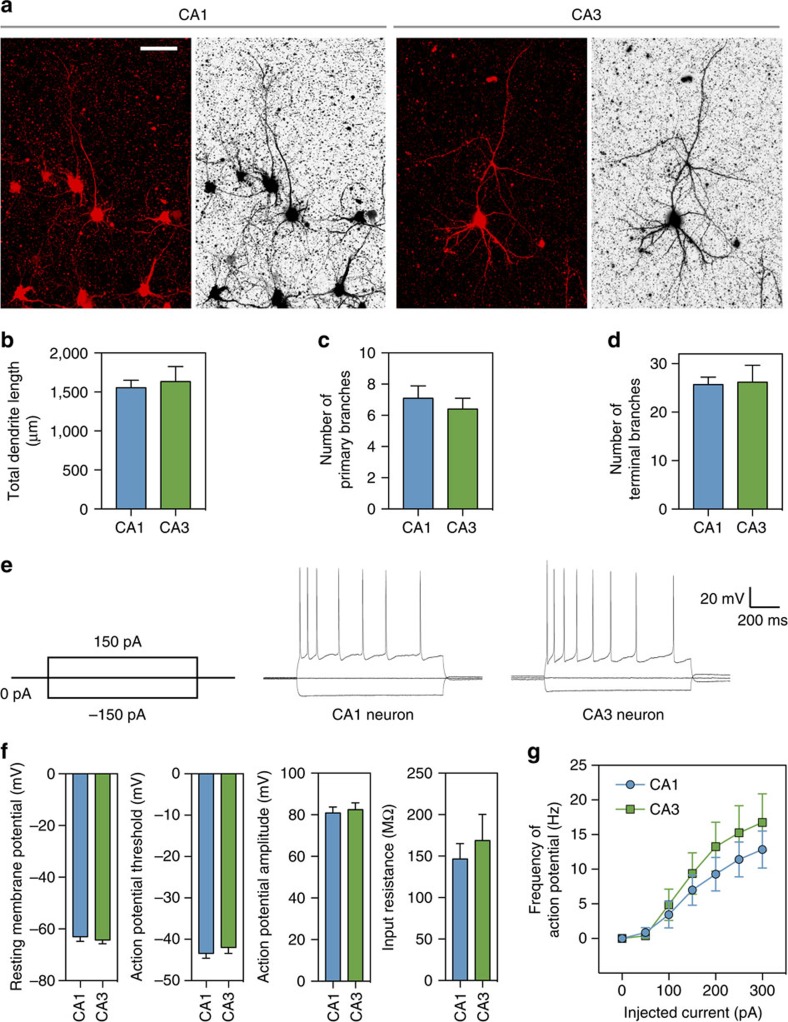
Morphological and electrophysiological properties of CA1 and CA3 neurons in the CA3–CA1 construct. (**a**) Representative confocal images (*z* stacked) of CA1 and CA3 neurons. Neurons in the CA3–CA1 construct were infected with adeno-associated virus (AAV)-mCherry for morphological analysis of CA1 and CA3 neurons. Fluorescence micrographs (left) and inverted images (right) are shown. Scale bar, 50 μm. (**b**–**d**) Analysis of dendrites of CA1 and CA3 neurons at DIV 21 in anisotropically organized CA3–CA1 culture. Quantification of total dendritic length (**b**), number of primary dendritic branches (**c**) and terminal branches (**d**) of CA1 and CA3 neurons are shown. Error bars indicate s.e. of mean (s.e.m.; *n*=10 each). (**e**) Representative traces of action potentials from a CA1 and a CA3 neuron in response to the current injection protocol (−150 or 150 pA steps) shown on left in anisotropically organized CA3–CA1 cultures. To allow easy penetration by a patch-clamp electrode, low concentration of collagenase was briefly treated (0.2 mg ml^−1^, 10 min). (**f**) Intrinsic electrophysiological properties of CA1 and CA3 neurons. (**g**) Firing rates of CA1 and CA3 neurons as a function of injected current. Error bars indicate s.e.m. (**f**,**g** *n*=7 for CA1 and *n*=8 for CA3).

In addition to extending long axons, CA1 and CA3 neurons elaborated dendritic structures in the anisotropically organized 3D collagen gels. To visualize the morphologies of individual dendrites, CA1 and CA3 neurons in the pre-stretched chips were infected with a low concentration of adeno-associated virus (AAV)-mCherry or AAV-EGFP for sparse labelling (Fig. 5a). When examined at DIV 21, we found that total dendritic length and the numbers of dendrites and dendritic branches (Fig. 5b–d) of CA1 and CA3 neurons were comparable to the values obtained from *in vivo* studies^46^,^47^,^48^,^49^,^50^. Altogether, these results suggest that CA1 and CA3 neurons develop normally in anisotropically organized gels and acquire morphological properties similar to those of native hippocampal neurons in the brain.

To examine intrinsic membrane properties of neurons in the 3D construct, we performed patch-clamp recordings from CA1 and CA3 neurons growing in directionally aligned collagen gels at DIV 21–28 (Fig. 5e–g). Under current-clamp configuration, CA1 and CA3 neurons generated action potentials in response to depolarizing current injections (Fig. 5e), similar to the characteristics of CA1 (refs 51, 52) and CA3 (refs 53, 54, 55) neurons in acute hippocampal slices. We also confirmed that other electrophysiological properties, such as resting membrane potential, action potential threshold and amplitude, and input resistance of CA1 and CA3 neurons (Fig. 5e,f), were comparable to the reported values from CA1 (refs 51, 52) and CA3 (refs 53, 54, 55) neurons in acute hippocampal slices. Under voltage-clamp configuration, spontaneous excitatory postsynaptic currents (sEPSCs) were detected in all recorded neurons, but only two cells showed spontaneous inhibitory postsynaptic currents (sIPSCs) among a total of eleven recorded cells (Supplementary Fig. 7).

**Figure 6:**
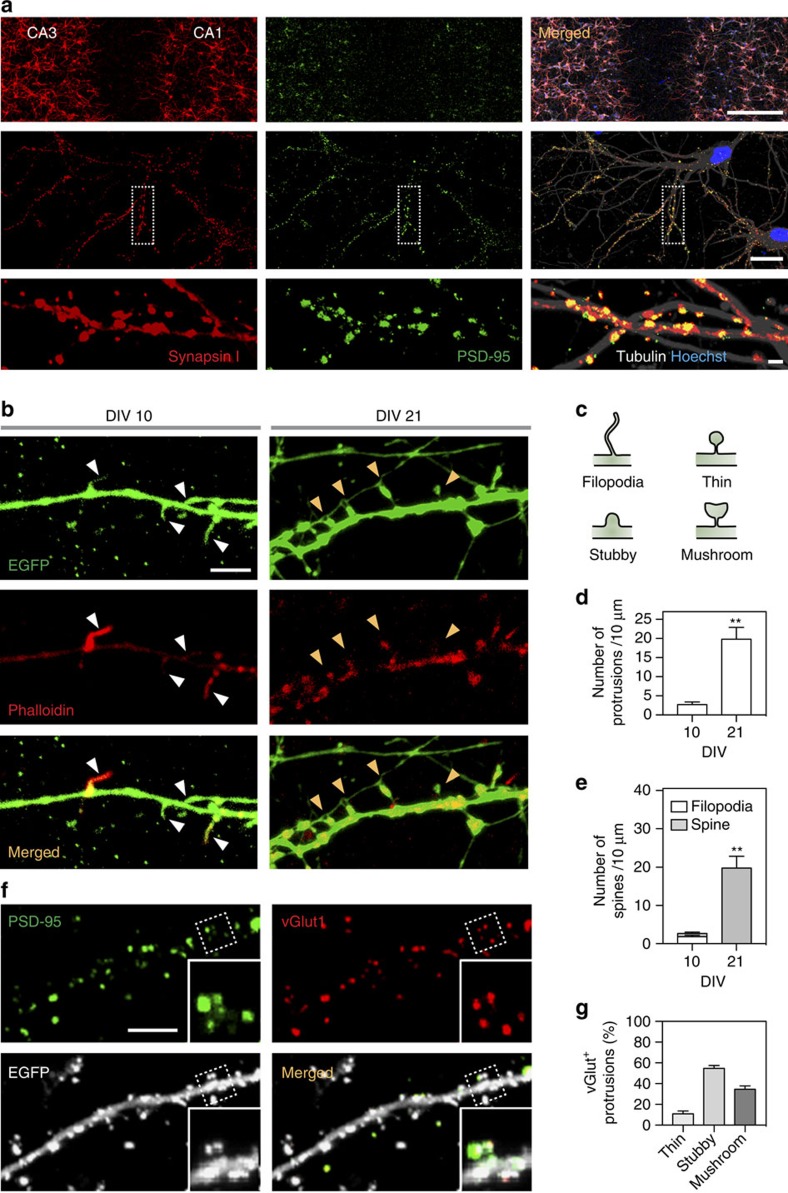
Analysis of spines in the CA3–CA1 construct. (**a**) Representative confocal fluorescence micrographs of the CA3–CA1 culture immunostained for synapsin-I, PSD-95, tubulin and nuclei (Hoechst). Boxed areas are enlarged and rotated at bottom panels. Scale bars, 500 μm (top), 20 μm (middle), 2 μm (bottom). (**b**–**f**) Neurons in the CA3–CA1 construct were infected with adeno-associated virus (AAV)-EGFP for morphological analyses. (**b**) Representative confocal images showing filopodia (white arrow heads) at DIV 10 and spines (yellow arrow heads) at DIV 21 in the CA1 region of the CA3–CA1 construct. Neurons were fixed at DIV 10 or DIV 21 and immunostained for actin (red, phalloidin). Scale bar, 5 μm. (**c**) Schematic drawings of spine morphologies. (**d**,**e**) Quantification of dendritic protrusion density (**d**) and type (**e**) at DIV 10 and DIV 21. Error bars indicate s.e. of mean (for 54≤*n*≤496 protrusions from 6 dendrites; s.d. of 1.651 and 7.628 for protrusions at DIV 10 and 21, respectively; s.d. of 0.9205 and 0 for filopodia at DIV 10 and 21, respectively; 0.8685 and 7.628 for spines at DIV 10 and 21, respectively). ** and ***P=3.04 × 10^−3^ and 1.48 × 10^−3^, respectively (parametric unpaired *t*-tests with Welch's correction assuming unequal s.d.). (**f**) Representative confocal fluorescence micrographs of the CA3–CA1 culture immunostained for PDS-95 and vGlut1. Boxed areas are enlarged at bottom right. Scale bars, 5 μm. (**g**) Analysis of vGlut^+^ spines (thin, stubby and mushroom-types) at DIV 21.

Immunolabelling experiments revealed that by DIV 21, presynaptic protein synapsin-I often colocalized with or located adjacent to postsynaptic protein PSD-95, suggesting proper development of synapses (Fig. 6a). To further examine synapse development in 3D constructs, individual neurons were labelled with AAV-EGFP and dendritic protrusions at DIV 10 and 21 were compared. At DIV 10, not many protrusions were observed and most protrusions were filopodia (Fig. 6b–e), characteristics of developing neurons^56^. The density of dendritic protrusions markedly increased by DIV 21 and quantification of vGlut^+^ presynaptic terminals revealed that most synapses were mature forms (Fig. 6f,g).

**Figure 7:**
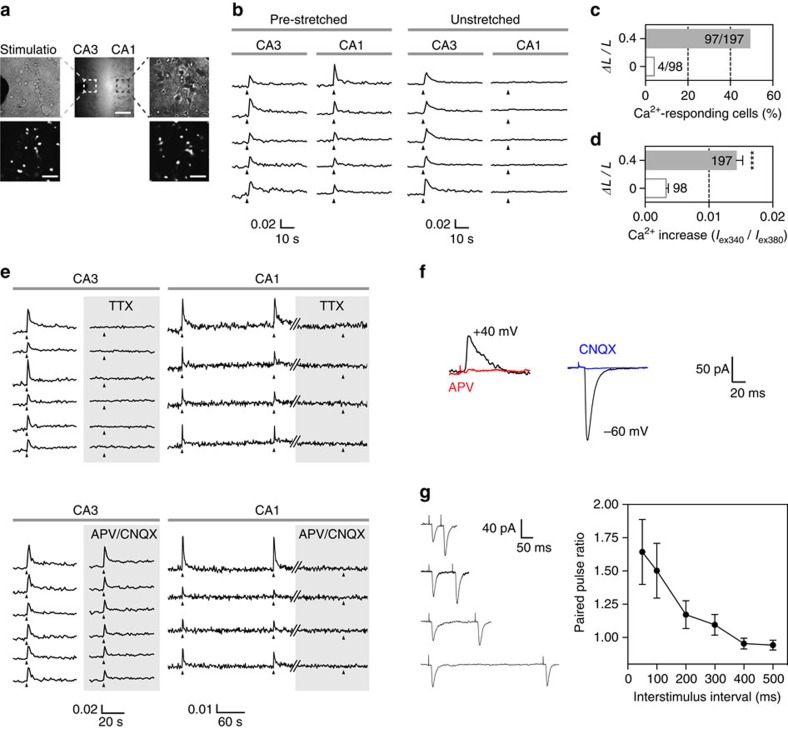
Establishment of a functional CA3–CA1 neural circuit in anisotropically organized gels. (**a**) A concentric bipolar electrode was placed in the CA3 region to stimulate neurons and recording pipette was placed in the CA1 region. Bright field (top) and fura-2/AM-loaded (bottom) images show the location of electrical stimulation and where Ca^2+^ responses were recorded from in CA3 and CA1 regions. Boxed areas are enlarged on left and right. Scale bars, 500 μm (upper bar), 100 μm (lower two bars). (**b**) Representative Ca^2+^ responses of fura-2/AM-loaded CA3 and CA1 neurons, grown either in pre-stretched (*ΔL*/*L*=0.4) or unstretched (*ΔL*/*L*=0) PDMS chips, as indicated. Triangles indicate electrical stimulation (1 s, 20 Hz). (**c**) Percentage of Ca^2+^-responding cells in the CA1 region growing either in pre-stretched (*ΔL*/*L*=0.4) or unstretched (*ΔL*/*L*=0) PDMS chips. Numbers of total and responding cells are indicated. (**d**) Average peak ratio of Ca^2+^ transients of CA1 neurons in pre-stretched (*ΔL*/*L*=0.4) or unstretched (*ΔL*/*L*=0) PDMS chips. Number of cells pooled from at least three independent recording sessions is indicated. Error bars indicate s.e. of mean (s.e.m.; s.d. of 0.003564 and 0.01437 for *ΔL*/*L*=0 and 0.4, respectively). ***P=5.83 × 10^−11^ (parametric unpaired *t*-test with Welch's correction assuming unequal s.d.). (**e**) Representative Ca^2+^ responses in CA3 and CA1 neurons evoked by repetitive electrical stimulation of CA3 neurons grown in pre-stretched (*ΔL*/*L*=0.4) PDMS chips. Triangles indicate electrical stimulation. Traces show Ca^2+^ responses in the absence or presence of TTX (left) or APV and CNQX (right) in CA3 and CA1 neurons, as indicated. (**f**) Representative evoked EPSC (eEPSC) recorded from a CA1 neuron. Top, NMDA receptor-mediated eEPSC at a holding potential of +40 mV before (black) and after (red) APV treatment. Bottom, AMPA receptor-mediated eEPSC at a holding potential of −60 mV before (black) and after (blue) CNQX treatment. (**g**) Left, Representative eEPSCs traces from CA1 neurons in response to CA3 paired-pulse stimuli at a 50, 100, 200 and 500 ms inter-stimulus interval. Right, Average values of paired-pulse ratio (amplitude ratio of the second to the first EPSC) from CA1 neurons plotted as a function of inter-stimulus interval. Error bars indicate s.e.m. (*n*=9 for each interval). For (**f**,**g**) low concentration of collagenase was briefly treated (0.2 mg ml^−1^, 10 min) to allow easy penetration by a patch-clamp electrode.

To assess functional synaptic connections in anisotropically aligned gels, we electrically stimulated CA3 neurons by placing a concentric bipolar electrode in the CA3 region, and recorded Ca^2+^ signals from CA1 neurons (Figs 4b and 7a). Ca^2+^ responses in the CA1 region, which were induced by synaptic transmission between CA3 and CA1 neurons, were readily detected only in the pre-stretched but not unstretched chips (Fig. 7b). By contrast, Ca^2+^ signals in the CA3 region, which were evoked by direct electrical stimulation, were detected in both platforms (Fig. 7b). The percentage of Ca^2+^-responding CA1 cells (Fig. 7c) and peak ratio of Ca^2+^ signals in CA1 neurons (Fig. 7d) were much higher in pre-stretched PDMS chips compared with unstretched chips, suggesting that aligned collagen scaffolds facilitate the formation of a functional neural network.

Both electrically- and synaptically-induced calcium responses were completely abolished by TTX, a potent inhibitor of voltage-gated Na^+^ channels (Fig. 7e, top). However, blockade of postsynaptic AMPA and NMDA receptors by treatment with CNQX and APV selectively inhibited the synaptically-induced Ca^2+^ responses in CA1 neurons without affecting the electrically-induced Ca^2+^ increases in CA3 neurons (Fig. 7e, bottom).

We also recorded evoked EPSCs (eEPSCs) in CA1 neurons to confirm synaptic connections between CA1 and CA3 neurons in the pre-stretched PDMS chip. We were able to obtain AMPA receptor-mediated eEPSC at a holding potential of −60 mV (abolished by treatment with CNQX) and NMDA receptor-mediated eEPSC at a holding potential of +40 mV (abolished by treatment with APV) in CA1 neurons by electrical stimulation of the CA3 region (Fig. 7f). When we recorded paired-pulse ratio (PPR) as a measure of short-term plasticity, the PPR profile of our 3D culture was comparable to previously studies from rat hippocampal slices^12^,^13^,^14^ (Fig. 7g).

Taken together, these results demonstrate that electrical stimulation of CA3 neurons evokes Ca^2+^ responses and postsynaptic currents in CA1 neurons, that synaptic transmission is mediated by postsynaptic AMPA and NMDA receptors and that synaptic connectivity is markedly enhanced by anisotropically organizing the fibrous scaffold. Similar results were obtained by culturing CA3 and CA1 mouse primary hippocampal neurons (Supplementary Fig. 8): a functional CA3–CA1 neural circuit was formed in 3D culture platforms and neural connectivity was augmented in directionally aligned gels.

To further support that organization of the scaffold is essential for the structural and functional connectivity, we induced fibril alignment perpendicular to the direction of intended CA3–CA1 connectivity (Supplementary Fig. 9). For this purpose, PDMS was pre-compressed along the *y* axis, followed by loading of cell-seeded and cell-free collagen solutions into the three compartments. After 5 min, strain was released and collagen-loaded PDMS was restored to original configuration (Supplementary Fig. 9a; see also Supplementary Fig. 2). As expected, alignment was induced along the *y* axis and axons did not grow across the axonal compartment (Supplementary Fig. 9a). For the pre-compression, we designed 10 mm-thick PDMS chips because 1 mm-thick chips (which were used for pre-stretch experiments) became distorted during the pre-compression step. However, the 10 mm-thick PDMS channels did not fit into our calcium imaging stage, preventing us from assessing functional connectivity. To circumvent this technical issue, we used the 1 mm-thick chips, but pre-stretched along the *x* axis (Supplementary Fig. 9b), as an alternative way to induce fibril alignment perpendicular to the direction of the intended CA3–CA1 connectivity. We confirmed that the alignment was induced as intended, that is, along the *y* axis, and axons failed to grow across the axonal compartment. Importantly, when CA3 neurons were electrically stimulated, little, if any, responses were detected from CA1 neurons (Supplementary Fig. 9c), showing lack of functional connections between the two neuronal populations. By contrast, Ca^2+^ signals in the CA3 region, which were evoked by direct electrical stimulation, were readily detected. These results further support the notion that anisotropic organization of the culture platform is important to achieve both structural and functional connectivity.

Hippocampal CA3–CA1 circuit has been extensively studied as a model system for investigating mechanisms of synaptic plasticity. To test if our 3D culture platform can be used for synaptic plasticity studies, we recorded eEPSCs from CA1 neurons in response to stimulation of CA3 neurons, as in acute slice preparations. A marked long-term plasticity was readily obtained, in the form of long-term depression (LTD), from CA1 neurons after stimulating the CA3 region with a low frequency stimulation protocol (LFS: 1 Hz, 15 min), which was blocked by APV (Supplementary Fig. 10). These results indicate that the anisotropically organized 3D culture provides a readily accessible platform to investigate synaptic plasticity.

### Assessment of general applicability of the alignment method

Our method for culturing hippocampal neurons dissociates CA3 and CA1 cells from perinatal embryos. At this stage, hippocampal tissue contains majorly neurons but also some astrocytes. In our culture, we observed that astrocytes in the pre-stretched but not the unstretched platform were aligned (Supplementary Fig. 11), similar to the alignment of neurons. To assess if this strategy could be applied to align other cell types, we cultured 3T3 fibroblasts and C2C12 myoblasts in pre-stretched and unstretched culture platforms (Supplementary Fig. 11). Both types of cells aligned along the *x* axis in pre-stretched PDMS chips, but oriented randomly in unstretched chips, demonstrating the general applicability of this alignment strategy.

## Discussion

Reconstruction of a physiologically relevant neural circuit that models *in vivo* connectivity is a major challenge in engineering equivalents of brain tissue. To reconstruct a structurally organized neural network in 3D that mimics *in vivo* connectivity, several design criteria should be considered: first, anisotropy to guide directional growth of axon tracts, second, modularity to model the compartmentalized nature of neural network, third, continuity to connect such compartments seamlessly, and finally innocuity to cause minimal damage to primary neurons. To the best of our knowledge, no single approach has satisfied all four criteria. By generating a contact guidance field to orient axon growth, here we have recapitulated the hippocampal Schaffer collateral pathway, the parallel axon tracts of CA3 neurons that project to area CA1, in a monolithic gel. Neurons cultured in the aligned platform exhibited electrophysiological properties similar to those of acute brain slices, and CA3 and CA1 neurons formed structural and functional synapses in 3D. Furthermore, functional connectivity was markedly enhanced by anisotropically organizing the culture scaffolds.

Our new approach offers several advantages. First, by exploiting the mechanical property of PDMS and kinetics of collagen fibrillogenesis, long-lasting alignment can be effectively induced by instantaneous strain. To this end, it is essential to apply (and release) strain within a critical window of time during fibrillogenesis. Second, as transient strain is sufficient for alignment, cells populated in the sol/gel biphasic state of collagen are exposed to minimal stress. Reducing mechanical stress is critical when dealing with sensitive cells, such as mammalian primary neurons, as exposure to strong or sustained stress can compromise cell viability and alter physiological properties. We confirmed that pre-deformation caused little cell death and that primary neurons that had been processed through the procedure to induce alignment underwent differentiation, formed connections, and showed long-term viability (up to at least 90 days) in culture. Developing a method that is compatible with mammalian primary neuronal culture is important not only to recapitulate certain neural circuitry of the mammalian brain and investigate high brain function (for example hippocampus-dependent synaptic plasticity and LTP/LTD), but also to fully exploit the power of existing and future mouse genetic tools, especially if one considers applying this method for studying dysfunction of neural circuits in neurological disorders. Third advantage is that this method does not require layer-by-layer assembly of individual modules to create a multi-modular conglomerate. Stacking individual components can place considerable design constraints and often generate undesired architectural seams in the final construct. For reconstruction of a neural circuit, structural continuity between juxtaposed compartments is essential: a neuron located in one compartment should extend an axon, which grows into another compartment, and reaches yet another compartment where dendrites of partner neurons reside. A growing axon should not be encountered by interstitial spaces that can present physical barriers to neurite outgrowth and network formation. In this study, collagen fibrils play a part to interconnect adjacent compartments that are constructed simultaneously. Fourth advantage is that this method produces homogeneous alignment. We confirmed that the angle of alignment was consistent throughout the depth of several hundred micrometres. Alignment induced by other methods, such as application of electric^16^,^17^ or magnetic^18^,^19^ fields or shear flow^19^,^21^,^22^,^57^,^58^, is inherently inhomogeneous as the degree and angle of alignment in a particular location reflect relative position of cells or scaffold materials in the driving force field, which becomes more evident in large 3D constructs. Fifth, this method induces alignment in cell-free and cell-laden parts simultaneously and independently of the presence of cells. Some methods exploit cell traction to deform the scaffold or fibrillar network as a way of inducing alignment^20^,^24^,^26^,^27^. Accordingly, alignment requires not only the existence of cells but also can depend on the type and density of cells^27^,^59^. Last but not least, this method is versatile in that in addition to alignment direction, the number and dimension of compartments as well as their composition (for example, density and types of cells in each compartment, combinations of cell-laden and cell-free compartments and so on) can be readily adjusted to meet different needs. Here, we used collagen type I because of its availability and cost, because it self-assembles into a long fibrous structure, and because many studies have demonstrated the technical feasibility and tunability of using collagen type I to create scaffolds for 3D culture^60^. However, as long as they elongate and bundle into fibrous structures from less ordered components, different types of collagen or collagen mimetics can also be used.

Emerging lines of evidence suggest that a number of psychiatric and neurological disorders result from abnormalities of neuronal circuits: malfunction of the cortico-striatal connection is implicated in Alzheimer's disease^61^, basal ganglia-thalamocortical circuits are disrupted in Parkinson's disease^62^ and hippocampal network aberrations are associated with epilepsy and schizophrenia^63^. Although neurological disorders affect a number of brain regions and produce a complex array of symptoms, basic phenotypes likely exist at the level of simple network. Our method can be adapted to reconstruct normal and diseased neural circuits and we anticipate that when blended with patient-derived stem cell technology, further insights may be gained towards understanding the dysfunction of neural circuits that underlie a wide range of brain disorders.

## Methods

### Preparation and labelling of collagen fibrils

For imaging of collagen fibrils shown in Figs 1b and 2b,g, and Supplementary Movie 1, collagen was conjugated with tetramethylrhodamine (TRITC; Life Technologies)^64^. Briefly, type I collagen was extracted by harvesting tendons from rat tails, solubilizing the tendons in 0.1% [w/v] acetic acid, purifying collagen with high-speed centrifuge (for example, 9,500 r.p.m.) at 4 °C, and lyophilizing. Then, a collagen solution (for example, 2 mg ml^−1^) in 0.1 M sodium bicarbonate buffer (pH 9), was mixed with 10 mg ml^−1^ TRITC in dimethyl sulfoxide (DMSO). Next, this mixture was agitated in dark continuously with a tube rotator at 4 °C for 24 h. After removing free TRITC molecules by dialysis in a bath of 0.1% [w/v] acetic acid at 4 °C, TRITC-conjugated collagen was lyophilized again before use. For imaging of collagen fibrils shown in Figs 1d and 4d and Supplementary Movies 2, 4, 5, 6 and 7, collagen fibrils were labelled with 5-(and-6)-carboxyte-tramethylrhodamine succinimidyl ester (TAMRA; Invitrogen) as reported elsewhere^65^. Briefly, 5 μM TAMRA in phosphate-buffered saline (PBS) was applied to collagen scaffolds. After incubating at room temperature for 1 h, the collagen scaffolds were rinsed three times with PBS before confocal laser scanning microscopy.

### Preparation of cell-free or cell-seeded collagen

Custom-extracted collagen described above was used for Figs 1b and 2b–e,g, and commercial type I collagen (8–11 mg ml^−1^, rat tail; Corning) was used for all other data. Neutralized (pH 7.5, determined by phenol red in the mixture) collagen solutions (2.5 mg ml^−1^) was prepared as follows: (1) either 110–160 mg or 110–160 μl (depending on the concentration of collagen stock solution) collagen stock in acetic acid (0.1% [w/v] for custom-extracted collagen and 0.02 N for commercial collagen) was transferred to a 1 ml microtube. (2) A total of 50 μl of 10 × Dulbecco Modified Eagle Medium (DMEM; Sigma-Aldrich) was added and gently mixed. (3) Overall, 10–20 μl of 0.5 N NaOH was added for neutralization (determined by phenol red in the mixture). (4) A total of 50 μl of 1 × DMEM (Lonza) or cell-suspended-DMEM was added, and gently mixed. 5) A total of 1 × DMEM was added to match a total volume of 500 μl. To prepare C2C12 myoblast-seeded collagen, the mixture was additionally supplemented with 50 μl of 10 mg ml^−1^ Matrigel (BD Biosciences). Seeding densities of cells in collagen were 2 × 10^6^ (two right panels in Fig. 4e and Supplementary Fig. 5) and 4 × 10^6^ (Figs 3, 4, 5, 6, 7) cell ml^−1^. Then, cell-free or cell-seeded collagen was immediately processed for gelation and alignment. All steps were performed on ice to minimize undesired gelation.

### Alignment of collagen fibrils by pre-deformation of PDMS

A PDMS (Sylgard 184, Dow Corning) block (1 or 10 mm-thick for pre-stretching or pre-compression, respectively), patterned with a rectangular well (for example, 3 (*x*) × 15 (*y*) × 0.35 (*z*) mm for pre-stretching and 5 × 5 × 0.4 mm for pre-stretching), was replicated from a duralumin master. This duralumin master was silanized with a fluorinated silane (trichloro(1H,1H,2H,2H-perfluorooctyl)silane; Sigma-Aldrich) before use. To promote the adhesion of collagen on PDMS, inner surfaces of the well were coated with polydopamine by applying 2 mg ml^−1^ dopamine hydrochloride dissolved in 10 mM Tris-HCl buffer (pH 8.5). After 2 h at room temperature, dopamine hydrochloride was aspirated and the well was rinsed three times with deionized (DI) water, followed by air-drying in a clean bench. The PDMS block was pre-deformed to a certain extent (from *L* to *L+ΔL* ) in *y* direction (Fig. 1a), using custom frames. The pre-deformed well was then loaded with either cell-free or cell-seeded collagen. After partial gelation for *t*_D_ (5 min, unless stated otherwise) at room temperature, the pre-deformed PDMS block was restored to original configuration (that is, from *L*±*ΔL* to *L*). Then, PDMS block was placed in a humidified incubator (37 °C and 5% CO_2_) to complete gelation for 30 min.

### Fabrication of an aligned, multi-modular 3D culture platform

A multichannel PDMS chip composed of three inlet channels (2.1 × 5 × 0.35 mm), a merging channel (2.1 × 15 × 0.35 mm), and a common outlet (Fig. 4a), was replicated from a silanized duralumin master. An anisotropically organized, multi-modular 3D culture platform was fabricated as follows (see Fig. 4a and Supplementary Fig. 4): (1) A reference frame (H-shape, to define *L*) and two clamping bars machined with poly(methyl methacrylate) (PMMA) were sterilized and placed in a clean bench. (2) The clamping bars were inserted into side grooves of the reference frame. (3) The multichannel PDMS chip was pre-coated with polydopamine for adhesion between collagen and PDMS and placed on the reference frame (channel side down). (4) The multichannel PDMS chip and the clamping bars were fastened by two clamps on the reference frame. (5) The reference frame was removed by disassembling the clamping bars. (6) The clamping bars were inserted into side grooves of a stretching frame (H-shape) to deform the PDMS chip (from *L* to *L*±*ΔL*). (7) To prevent undesired bubble formation in the channels, cell-free collagen was injected from the outlet into bottom of the three inlets (optional). Cell-free and neuron-seeded collagen solutions were prepared as described above. (8) A total of ∼30 μl of cell-free collagen was loaded into a reservoir of the inlet in the middle (inlet 2). (9) A total of ∼30 μl of CA1 neuron-seeded collagen was loaded into a reservoir of inlet 1. (10) A total of ∼30 μl of CA3 neuron-seeded collagen was loaded into a reservoir of inlet 3. (11) Collagen solutions loaded into three inlets filled the merging channel by withdrawing from a common outlet at a speed of ∼7.3 mm s^−1^. (12) Partial gelation was allowed for optimal *t*_D_ (5 min) at room temperature. (13) Strain was released by disassembling a clamp and a clamping bar. (14) After disassembling the other clamp and the clamping bar, gelation was completed in a humidified incubator (37 °C and 5% CO_2_) for 30 min. (15) The multichannel PDMS chip was transferred to a Petri dish (channel side up). (16) A total of 5 ml of culture media was added for culture.

### Live/dead cell viability assay

Cell viability was assessed by incubating samples in PBS containing 0.5 μg ml^−1^ calcein-acetoxymethyl (calcein-AM; Molecular probe) and 2 μg ml^−1^ propidium iodide (PI; Sigma-Aldrich) for 40 min at 37 °C and 5% CO_2_. After washing with PBS, confocal laser scanning microscopy (see below) was performed to count live and dead cells, which produced green and red fluorescence, respectively.

### MTS assay

Cells were assayed for 3-(4,5-dimethylthiazol-2-yl)-5-(3-carboxymethoxyphenyl)-2-(4-sulfophenyl)-2H-tetrazolium (MTS) reduction using the CellTiter 96 AQ_ueous_ One Solution Cell Proliferation Assay (Promega). Briefly, 4 × 10^6^ cell ml^−1^ of hippocampal neurons mixed in the collagen solution was loaded into the PDMS chip. After 2 h and 3 days, 20 μl of the MTS solution was added, incubated for 4 h at 37°C in a humidified 5% CO_2_ incubator, and absorbance was read at 490 nm.

### Immunofluorescence staining

To stain neurites, 3D constructs were fixed following a protocol for the preservation of cytoskeletal structures^66^ and blocking was performed with 2% [w/v] bovine serum albumin (BSA) in cytoskeleton-stabilizing buffer [60 mM 1,4-piperazinediethanesulfonic acid, 25 mM 4-(2-hydroxyethyl)-1-piperazineethanesulfonic acid (HEPES), 10 mM ethylene glycol tetraacetic acid (EGTA) and 2 mM MgCl_2_, pH 6.9] containing 0.1% [w/v] Triton X-100 for 2 h. To stain synaptic proteins, 3D constructs were fixed in 4% [w/v] paraformaldehyde (PFA) and blocking was performed with 2% [w/v] BSA in PBS. All steps were performed at room temperature. 3D constructs were then incubated sequentially with primary and secondary antibodies diluted in the blocking solution at 4 °C overnight. The following primary antibodies were used: mouse anti-βIII-tubulin (TuJ1) (1:1,000; Sigma; T8578), mouse anti-pan-axonal neurofilament marker SMI312 (1:1,500; Covance; SMI-312R), chicken anti-microtubule-associated protein 2 (MAP2) (1:500; Abcam; ab5392), rabbit anti-synapsin-I (1:2,000; Cell Signaling; #5297), guinea pig anti-vGlut-1 (1:1000, Millipore, AB5905), and mouse anti-PSD-95 (1:1,000, Thermo Fisher Scientific; MA1-045). Alexa Fluor conjugates (Alexa Fluor 488, 594, and 647) (1:1,000; Molecular Probes; A11001, 11012, A11042, A11076, A21236, A21247) were used for secondary antibodies. Filopodia were stained with Alexa Flour 594 Phalliodin (1:25; Molecular Probes; A12381). Nuclei were stained with Hoechst 33342 (1: 5,000; Molecular Probes; H3570). All samples were rinsed with PBS between the incubation steps.

### Confocal laser scanning microscopy

Fluorescence images were acquired using an inverted confocal laser scanning microscope (LSM 700; Carl Zeiss) equipped with solid-state lasers (405, 488, 555 and 639 nm). Post-image processing such as maximum intensity projection, orthogonal view, 3D reconstruction was performed using ZEN 2012 software (Carl Zeiss). For imaging of TRITC or TAMRA-labelled collagen fibrils (*xy*, *xz* and *yz* planes in Fig. 2b), scaffolds were placed on 170 μm-thick glass slides and covered fully with PBS droplets. *Z* stacked images (stack size, 50 μm; step size, 1 μm) were acquired with a × 20 objective (numerical aperture (NA) 0.6) to reconstruct single composite images. All other images of collagen fibrils were obtained by snapping single *xy* planes with a × 40 objective (NA 1.15; water immersion). For the live/dead assay, *z* stacked images (stack size, 200 μm; step size, 20 μm) in 2 channels (green for calcein-AM and red for PI) were acquired simultaneously with a × 20 objective (NA 0.3) to create maximum intensity projection images. For imaging of immunostained neurites (Fig. 3e), 3D constructs were mounted on 170 μm-thick glass slides using polyvinyl alcohol mounting medium (Sigma-Aldrich). Then, *z* stacked images (stack size, 30 μm; step size, 1 μm) were acquired with a × 10 objective to create maximum intensity projection images. For imaging of CA3–CA1 networks (left panel in Fig. 4e and Fig. 4f), tile scan and *z* stack imaging with a × 10 objective were performed simultaneously to acquire three horizontally stitched *z* stacked images (1,919.27 μm × 638.92 μm × 100 μm) in 2 or 3 channels, as indicated. For imaging of axons (two right panels in Fig. 4e), *z* stacked images (stack size, 100 μm; step size, 1 μm) were acquired with a × 40 objective to reconstruct 3D images. For imaging of pre- and post-synaptic markers and neurites (Fig. 6a), *z* stacked images (stack size, 50–65 μm; step size: 0.5 μm) in four channels were acquired simultaneously with a × 40 objective to create maximum intensity projection images.

### Measurement of orientations of collagen fibrils and cells

OrientationJ plug-in of ImageJ software was used for quantitative analyses of confocal fluorescence images. OrientationJ computed local orientations of each pixel in the confocal images. These local orientations were encoded in colours and displayed directly on the images. Single *xy* plane confocal images of TRITC- or TAMRA-labelled collagen scaffolds were used to analyse orientations of collagen fibrils (Figs 1b,d and 2c–e,g), and maximum intensity projection images from *z* stacked images were used to analyse orientation of cells and neurites (Figs 3c and 4f and Supplementary Figs 5a, 6b, 8a,c,e). Both colour-mapped images and local orientation frequencies with angular range from −90° to 90° were acquired by OrientationJ. Then, OI was calculated with the frequency data, using the following equation:


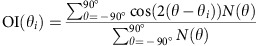


where *θ*_*i*_ is the alignment angle of interest, *θ* is local orientation angle, and *N*(*θ*) is number of pixels (frequency) at a given local orientation angle. We defined *x* direction as 0° (Fig. 1a) and evaluated orientation of alignments relative to *x* direction (that is, *θ*_*i*_=0). Therefore, OI can be re-written as


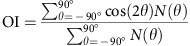


OI of 1, 0 and −1 indicate perfect alignment along *x* direction, random orientation, and perfect alignment along *y* direction, respectively. Polar frequency histograms of collagen fibrils or cells were generated by plotting normalized frequencies (that is, 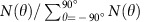) from the angular range (Origin Pro; Origin Lab). The angular colour scale was identical to the colour-coding of OrientationJ. An upper limit of the radial direction in all polar frequency histograms was fixed as 0.03 for better visibility.

### Isolation and culture of primary hippocampal neurons

Pregnant Sparague Dawley rats or ICR mice (E18.5) were purchased from DBL (Eumseong, Korea) and sacrificed for primary culture of hippocampal neurons, following a previous protocol^67^. All procedures were conducted according to the animal welfare guidelines approved by Institutional Animal Care and Use Committee of the Korea Institute of Science and Technology. Briefly, embryos were decapitated and the entire hippocampi were dissected out as an intact structure from the overlying neocortex for further subregion dissection. The boundaries of the DG were clearly visible under the dissection microscope. The CA1 or top portion of Ammon's horn was isolated at the division of the hippocampal fissure separating CA1 and DG/CA3. Using DG rostral and ventral ends as landmarks, cuts were made along the DG/CA3 boundary to isolate CA1 and DG/CA3. The hippocampi were treated with papain (Worthington) and serially triturated. Dissociated cells were counted and seeded at a density of 4 × 10^6^ cells ml^−1^ in a collagen mixture. Cells were cultured in plating medium consisting of neurobasal media supplemented with 5% fetal bovine serum (GIBCO), 2% B27-supplement (Invitrogen), 2 mM Glutamax-I (GIBCO) and 1% penicillin-streptomycin (GIBCO). After 1 day, plating medium was replaced by serum-free medium and maintained at 37 °C in a 5% CO_2_ humidified incubator. One-half of the medium was replaced with fresh culture medium every 2–3 days.

### Quantitative real-time polymerase chain reaction

For quantitative real-time polymerase chain reaction (qRT-PCR), total RNA was isolated from freshly dissected neurons from CA3 and CA1 with the TRI Reagent (Life technologies), and RNA was reverse transcribed by using Moloney murine leukaemia virus reverse transcriptase (ImProm-II Reverse transcription system; Promega). For quantification of mRNA, real-time PCR was performed by using SYBR Green Master mix (TAKARA) and ABI step one plus machine (Applied Biosystems). The sequences of the forward and reverse primers for rat *scip* and *ka1* pairs were F; 5′-tccctttctcttcccctctc-3′, R;5′-ggctctggtaaaacgaaacg-3′ and F;5′-gctccagcatgaccttcttc-3′, R;5′-ccctcctctgtgctcttcac-3′ and mouse *scip* and *ka1* pairs were F; 5′-agttcgccaagcagttcaag-3′, R;5′-tggtctgcgagaacacgtta-3′ and F;5′-aatgggtttcagcagattgg-3′, R;5′-accagggtggtgttgaagag-3′.

### Dendrite and spine analyses

For analysis of dendrites and dendritic spines in the CA3–CA1 culture, 3D constructs were infected with adeno-associated virus (AAV)-CamKIIα-mCherry or AAV-CamKIIα-EGFP (KIST virus facility). Cultures were fixed in 4% [w/v] PFA and washed in PBS. For spine analysis, 3D constructs were stained with PSD-95 and vGlut1. Consecutive stacks of images of dendrites (stack size, 45–80 μm; step size, 1 μm) were acquired with a × 20 objective (numerical aperture (NA) 0.6) and × 0.7 optical zoom, and those of spines (stack size, 4–12 μm; step size, 0.42 μm) were acquired with a × 40 objective (NA 1.15; water immersion) and × 5 optical zoom. For analysis of dendrites and spines, serial image files corresponding to *z* stacks of dendrite (45–80 optical sections per dendrite) and spine (10–30 optical section per spine) were directly processed with NeuronStudio ( http://research.mssm.edu/cnic/tools-ns.html), a software used for automated 3D neuron detection and analysis^68^,^69^,^70^. Voxel size of a dendrite was *x*=0.26, *y*=0.26, *z*=1.0 μm, and that of a spine was *x*=0.11, *y*=0.11, *z*=0.42 μm. For analysis of total dendritic lengths and branch numbers, dendritic trees were traced using a semi-automatic neurite tracer using a *xy* slice viewer to aid 3D visualization. To analyse the density and morphology of spines, 40∼50 μm dendritic segments were randomly selected and analysed. For spine classification, Neuronstudio was used based on default settings. After modelling of the dendrite surface, protrusions with a minimum volume of 5 voxels (0.020 μm^3^), a maximal width of 3 μm and a length of between 0.2 and 2 μm were retained as spines. Spines with a minimum head versus neck ratio of 1:1 and a minimum head diameter of 0.35 μm were classified as mushroom spines. Non-mushroom spines with a minimum volume of 10 voxels (0.040 μm^3^) were classified as stubby spines. All other spines were considered thin-type. After Neuronstudio processing, all spines were manually inspected and errors were corrected by an investigator blinded to experimental conditions.

### Electrophysiology

Electrophysiological recordings were performed with 3D constructs at DIV 21–28. Whole-cell patch-clamp recordings were achieved by applying collagenase type IA (0.2 mg ml^−1^; Sigma-Aldrich) at room temperature for 10 min, followed by inserting a patch electrode into the collagenase-treated 3D constructs. Recordings were performed at room temperature of 20–22 °C in artificial cerebral spinal fluid (aCSF) containing (in mM): 130 NaCl, 2.5 KCl, 26 NaHCO_3_, 1.25 NaH_2_PO_4_, 1.5 CaCl_2_, 1.5 MgCl_2_ and 10 D-glucose, oxygenated with 95% O_2_–5% CO_2_, at pH 7.4. Patch pipettes were pulled with a two-stage vertical puller (PC-10 Narishige). Resistance of the patch electrode was 5–10 MΩ. Visually guided whole-cell patch recordings were obtained using Axopatch 200A amplifier (Axon instruments) controlled by Clampex 10.0 software via a Digidata 1322A data acquisition system (Molecular devices), and analysed using pCLAMP 10 (Molecular devices). Action potential discharges were evoked under current-clamp conditions by long (1 s) current pulse injection with the patch electrode filled with an internal solution containing (in mM): 140 K-gluconate, 10 HEPES, 7 NaCl, 4 Mg-ATP and 0.3 Na_3_-GTP (pH adjusted to 7.3 with CsOH). To study frequency of action potentials in neurons, we used 1 s depolarizing pulses in 50 pA steps, in the current-clamp mode. Voltage-clamp recordings were obtained at a holding potential of −60 mV. Spontaneous IPSPs (sIPSPs) were measured from recordings of 4–5 min duration. Synaptic responses in CA1 neurons were evoked by 0.1 Hz stimulation of CA3 neurons (100 ms duration; 100–1,000 μA intensity) with a constant current isolation unit. To induce long-term depression (LTD), CA3 neurons were electrically stimulated by a repetitive low frequency stimulation protocol (1 Hz, 900 s duration; 100–1,000 μA intensity) and evoked EPSC (eEPSC) was recorded in CA1 neurons. Stimulus intensity was adjusted to evoke an EPSP or EPSC of ∼30–40% of the maximal amplitude. Pipette solution for spontaneous, evoked EPSP (eEPSP) and eEPSC recordings contained (in mM): 140 Cs-methanesulphonate, 8 NaCl, 1 MgCl_2_, 0.5 EGTA, 10 HEPES, 7 phosphocreatine di(tris) salt, 4 Mg-ATP, 0.3 Na_2_-GTP, 5 QX314 (pH adjusted to 7.3 with NMDG). The paired-pulse ratio was determined for six inter-stimulus intervals (50, 100, 200, 300, 400 and 500 ms) contiguously and the ratio of peak amplitude of the second EPSC to the first EPSC was calculated. To characterize pharmacology of eEPSC and LTD, CNQX (20 μM, Tocris) or APV (50 μM, Tocris) was administered to the culture in the aCSF recording solution. Pipette solution for sIPSC recordings contained (in mM):135 CsCl, 10 HEPES, 5 EGTA, 4 NaCl, 0.5 CaCl_2_, 4 Mg-ATP, 0.3 Na_2_-GTP and 5 QX314 (pH adjusted to 7.3 with CsOH). sIPSCs were pharmacologically isolated from EPSCs by addition of CNQX (20 μM) and APV (50 μM) in the aCSF recording solution.

### Calcium imaging

For Ca^2+^ imaging, 3D constructs at DIV 21–28 were incubated with 5 μM Fura-2 AM, 1 μM pluronic acid (Life Technologies) in aCSF for 30 min at room temperature and subsequently transferred to a microscope stage for imaging in aCSF. Regions of soma were selected as regions of interest (ROIs) and the images were captured at 1 Hz using iXon electron-multiplying cooled-coupled device (EM-CCD) camera (DV887 DCS-BV, ANDOR Technology), a filter wheel (Lambda 10-2, Sutter Instrument), and a xenon lamp (Lambda LB-LS 17, Sutter Instrument). Intensity images of 510 nm wavelength were taken at 340 and 380 nm excitation wavelengths with a filter wheel (Lambda 10-2, Sutter Instrument) and a xenon lamp, and the two resulting image sets were taken for ratio calculations. Imaging Workbench software (INDEC BioSystems) was used for acquisition of intensity images and conversion to ratios. Concentric bipolar electrodes (FHC) were placed in the cellular region (Figs 4b and 6e) of the 3D construct and 20 Hz stimulation (1 s duration; 100 μA intensity) was delivered through an A365 stimulus isolator (World Precision Instruments). To characterize pharmacology of Ca^2+^ transients, TTX (500 nM, Tocris) or CNQX (20 μM) and APV (50 μM) were administered to the culture in the aCSF recording solution.

### Statistical analysis

Statistical significance was determined from a minimum of three independent experiments. All statistical analyses were performed with Prism (GraphPad Software). Before determining statistical significance, Shapiro–Wilk test was performed to assess normality. ANOVA was used to compare the means of more than two samples or groups, and *t*-test was used to compare the means between two samples. One-way ANOVA with Tukey's multiple comparisons test was used to compare orientation indices of TRITC or TAMRA-labelled collagen fibrils, or neurites with varying *L*/*ΔL* (Figs 1c,e and 3f). Two-way ANOVA with Sidak's multiple comparisons test was used to compare relative absorbance at 490 nm from the MTS assays (Fig. 3d). Parametric unpaired *t-*test with Welch's correction assuming unequal s.d. was used to determine statistical significance in all other data (Figs 6d,e and 7d). **, *** and **** denote *P*<0.01, 0.001 and 0.0001, respectively, and calculated *P*-values are also specified.

### Data availability

The data that support the findings of this study are available within the article and its Supplementary Information files or from the corresponding authors on request.

## Additional information

**How to cite this article:** Kim, S. H. *et al*. Anisotropically organized three-dimensional culture platform for reconstruction of a hippocampal neural network. *Nat. Commun.* **8,** 14346 doi: 10.1038/ncomms14346 (2017).

**Publisher's note:** Springer Nature remains neutral with regard to jurisdictional claims in published maps and institutional affiliations.

## Supplementary Material

Supplementary Information Supplementary Figures.

Supplementary Movie 1 Time-lapse video showing the formation of collagen fibrils in a predeformed PDMS chip before and after releasing strain.

Supplementary Movie 2 Optical cross-sections of confocal fluorescence micrographs from an acellular, pre-stretched PDMS chip. Shown are TAMRA-labelled collagen fibrils in a region (160 μm × 160 μm × 100 μm) of the axonal compartment. Direction of collagen alignment is color-coded and angular color codes are identical to Fig. 1b.

Supplementary Movie 3 3D-rendered optical sections of confocal fluorescence micrographs for the CA3-CA1 culture (DIV 21) presented in Fig. 4e

Supplementary Movie 4 Optical cross-sections of confocal fluorescence micrographs for collagen fibrils and axons in a pre-stretched PDMS chip. Collagen fibrils and axons were labelled with TAMRA (red) and SMI312 (green), respectively. Shown is a region in the axonal compartment (160 μm × 160 μm × 100 μm). SMI312 confocal images were used to render 3D volume images presented in Fig. 4e.

Supplementary Movie 5 Optical cross-sections of confocal fluorescence micrographs for TAMRA-labelled collagen fibrils in a pre-stretched PDMS chip. Shown is a region in the axonal compartment (160 μm × 160 μm × 100 μm). Direction of collagen alignment is color-coded and angular color codes are identical to Fig. 1b.

Supplementary Movie 6 Optical cross-sections of confocal fluorescence micrographs for TAMRA-labelled collagen fibrils in a control, un-stretched PDMS chip. Shown is a region in the axonal compartment (160 μm × 160 μm × 100 μm). Direction of collagen alignment is color-coded and angular color codes are identical to Fig. 1b.

Supplementary Movie 7 Optical cross-sections of confocal fluorescence micrographs for collagen fibrils and axons in a control, un-stretched PDMS chip. Collagen fibrils and axons were labelled with TAMRA (red) and SMI312 (green), respectively. Shown is a region in the axonal compartment (160 μm × 160 μm × 100 μm). SMI312 confocal images were used to render 3D volume images presented in Fig. 4e.
