# Supplementary material for: Anisotropically organized three-dimensional culture platform for reconstruction of a hippocampal neural network
Source: Nat Commun. 2017 Feb 1;8:14346. doi: 10.1038/ncomms14346 (PMC5296669; doi:10.1038/ncomms14346)
Supplement: Supplementary Information — Supplementary Figures. [file ncomms14346-s1.pdf]

Supplementary Figures

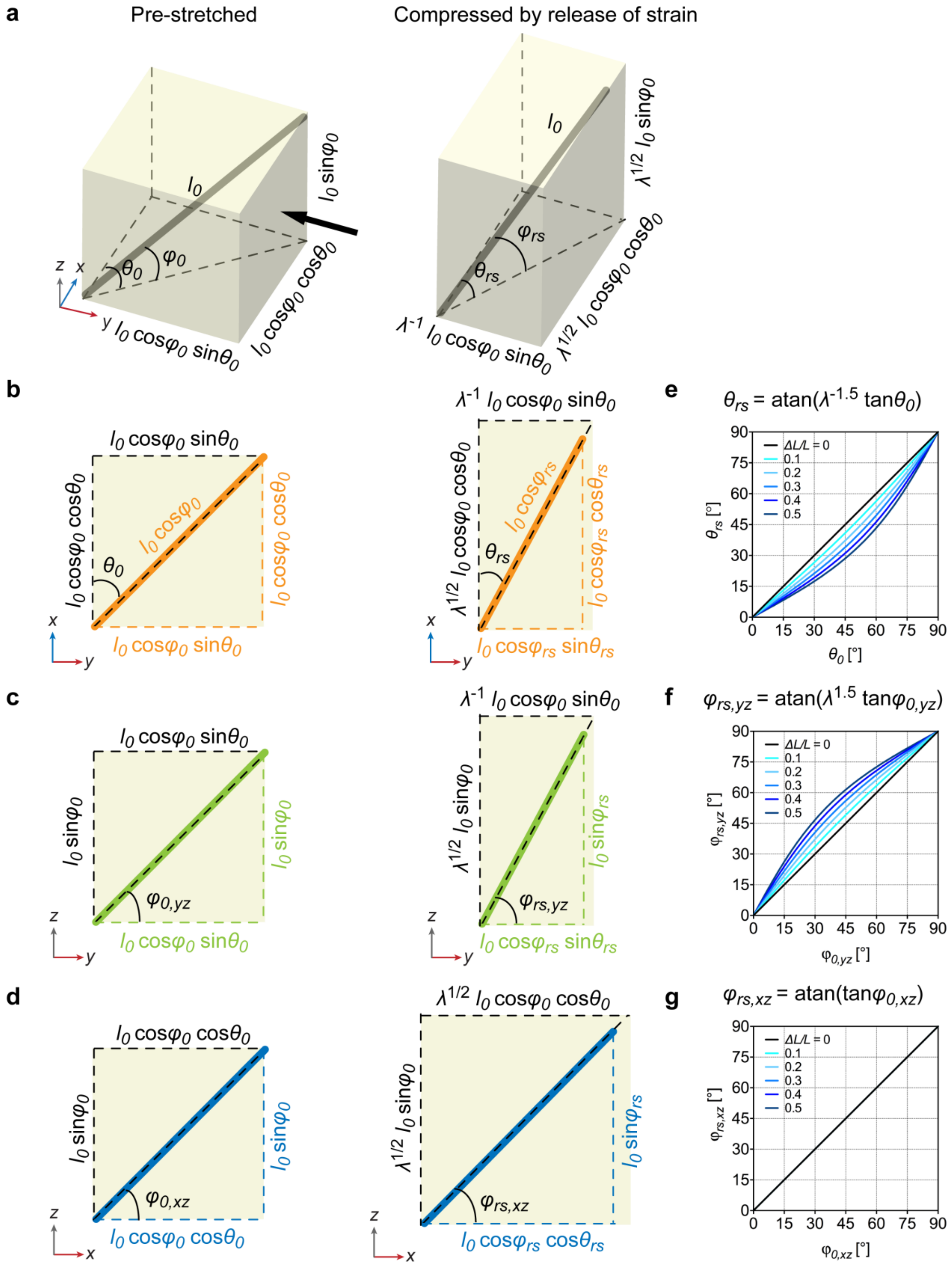

Supplementary Figure 1. Analytical model for anisotropic orientation of collagen fibrils in 3D matrix (pre-stretching). a, Schematic illustrating a collagen fibril (dark gray line) in a 3D viscoelastic matrix (cube) that is pre-stretched (*left*) and deformed by release of strain (*right*). Black arrow indicates the direction of axial strain exerted along *y*-axis in order to restore the pre-deformed matrix into original configuration. l_0 depicts length of a collagen fibril. θ and φ denote azimuthal and polar angles of the collagen fibril, respectively. λ is the magnitude of non-linear elastic deformation defined as $1+\varepsilon_{\mathrm{pre}}$, where $\varepsilon_{\mathrm{pre}}$ is the magnitude of strain (i.e., $\Delta L/L$). Subscriptions of *0* and *rs* denote pre-deformed and restored states, respectively. **b-d,** Orientation of the collagen fibril before (*left*) and after (*right*) release of strain is projected on *xy* (**b**; orange), *yz* (**c**; green), and *xz* (**d**; blue) planes. **e-g,** Plots of azimuthal and polar angles after releasing strain corresponding to illustrations presented on left (**b-d)**. Values were calculated from analytical models shown on left for each plot.

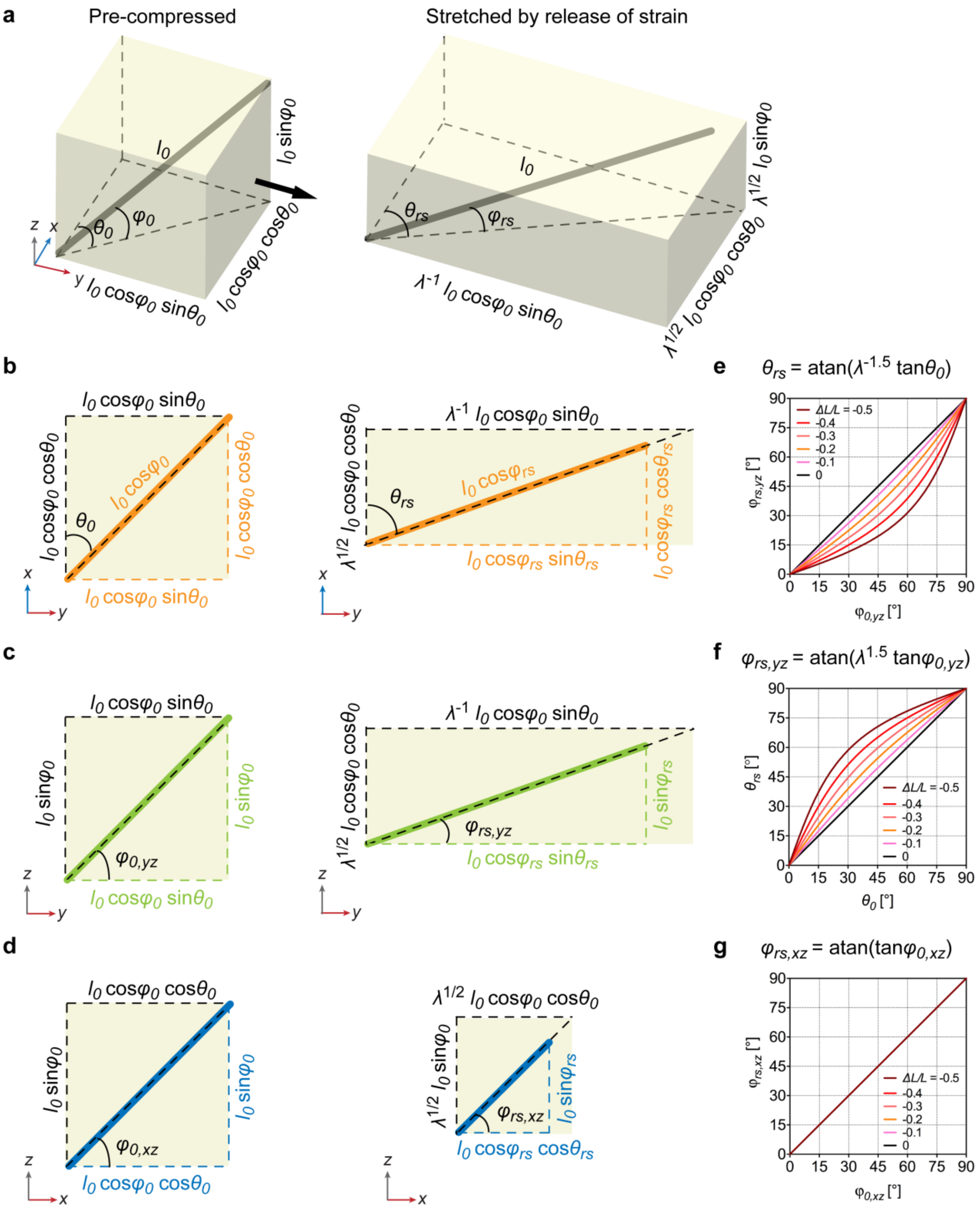

Supplementary Figure 2. Analytical model for anisotropic orientation of collagen fibrils in 3D matrix (pre-compression). a, Schematic illustrating a collagen fibril (dark gray line) in a 3D

viscoelastic matrix (cube) that is pre-compressed (*left*) and deformed by release of strain (*right*). Black arrow indicates the direction of axial strain exerted along *y*-axis in order to restore the pre-deformed matrix into original configuration. l_0 depicts length of a collagen fibril. θ and φ denote azimuthal and polar angles of the collagen fibril, respectively. λ is the magnitude of non-linear elastic deformation defined as $1+\varepsilon_{\mathrm{pre}}$, where $\varepsilon_{\mathrm{pre}}$ is the magnitude of strain (i.e., $\Delta L/L$). Subscriptions of *0* and *rs* denote pre-deformed and restored states, respectively. **b-d,** Orientation of the collagen fibril before (*left*) and after (*right*) release of strain is projected on *xy* (**b**; orange), *yz* (**c**; green), and *xz* (**d**; blue) planes. **e-g,** Plots of azimuthal and polar angles after releasing strain corresponding to illustrations presented on left (**b-d**). Values were calculated from analytical models shown on left for each plot.

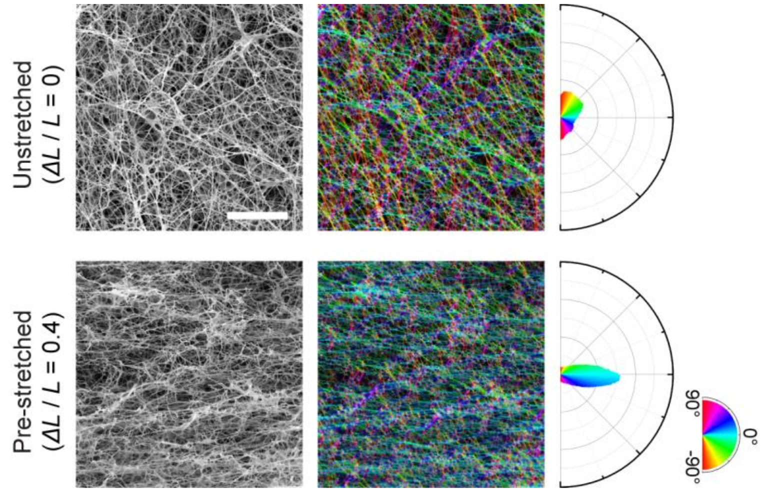

Supplementary Figure 3. **Scanning electron microscope images of collagen fibrils.** Shown are electron micrographs (*left*), color-mapped micrographs (*middle*), and polar frequency histograms (*right*) of collagen fibrils in unstretched (*top*; $\Delta L/L$=0) and pre-stretched (*bottom*; $\Delta L/L$=0.4) PDMS chips. Angular color scales for images and polar frequency histograms are identical, and semicircular color index for both is presented in the bottom right corner. Scale bar, 10 μm.

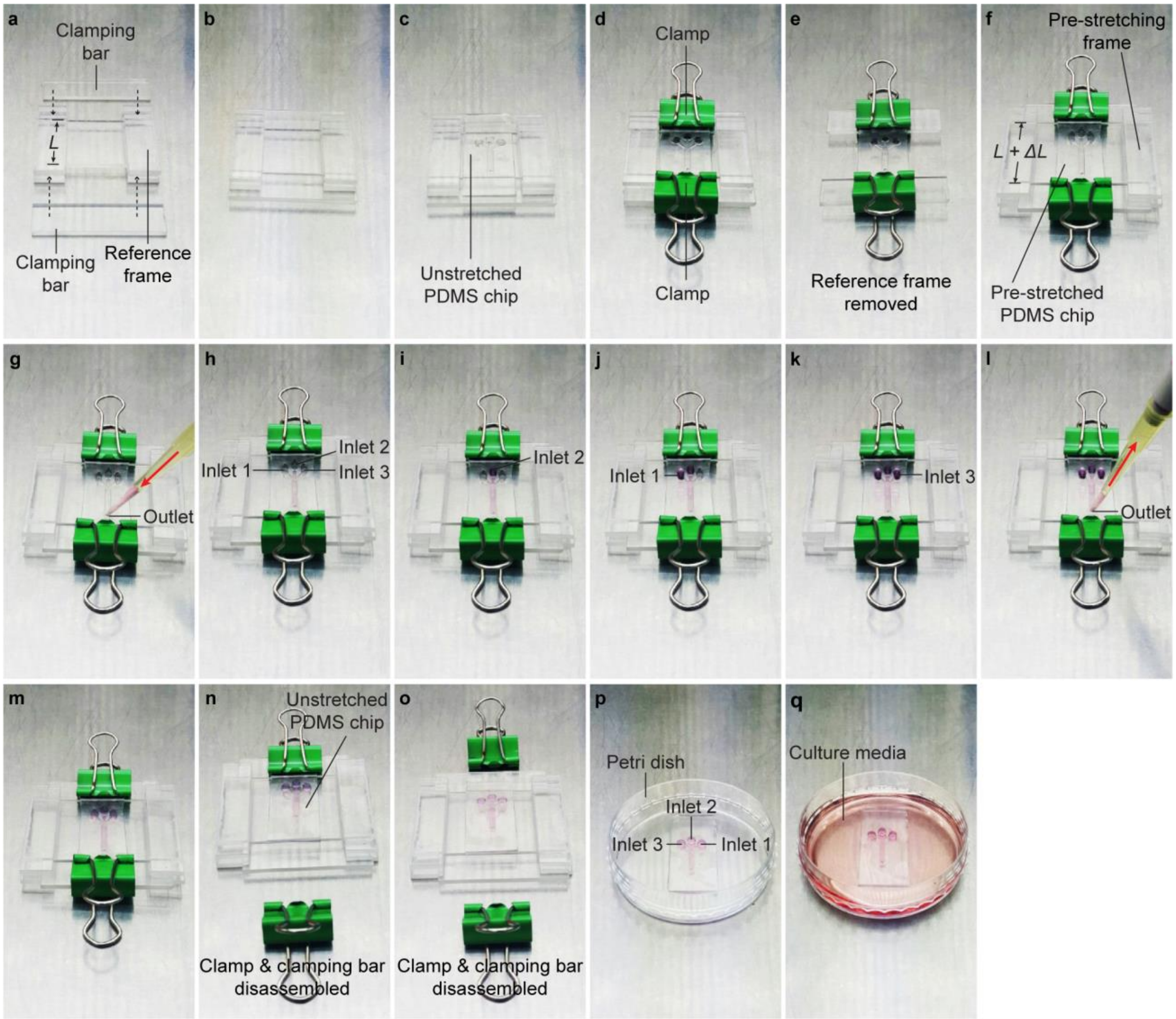

Supplementary Figure 4. **Detailed procedure for fabrication of an anisotropically organized, multimodular 3D culture platform.** Photographs of sequential steps to fabricate a hippocampal neural network using a three-channel PDMS chip by applying axial strain (pre-stretching). **a,** Two clamping bars and a reference frame (H-shape) to define *L*. **b,** The clamping bars inserted into side grooves of the reference frame. **c,** Placement of an unstretched PDMS chip. **d,** Fastening of the clamping bars, reference frame, and unstretched PDMS chip with two clamps. **e,** Removal of the reference frame. **f,** Assembly of the PDMS chip clamped with the clamping bars into side grooves of pre-stretching frame (H-shape) to deform the PDMS chip (from *L* to

L+*ΔL*). **g,** Injection of a cell-free collagen solution (*optional*, to prevent bubble formation). **h,** Cell-free collagen filled to bottom of inlets 1, 2, and 3. **i,** Loading of cell-free collagen into a reservoir of inlet 2. **j,** Loading of CA1 neuron-seeded collagen into a reservoir of inlet 1. **k,** Loading of CA3 neuron-seeded collagen into a reservoir of inlet 3. **l,** Withdrawal from outlet. **m,** Partial gelation for optimal t_D (5 min). **n,** Release of strain, followed by complete gelation. **o,** Disassembly of clamp and clamping bar. **p,** Placement of the anisotropically organized, multimodular 3D culture chip in a Petri dish. **q,** Application of media for further culture.

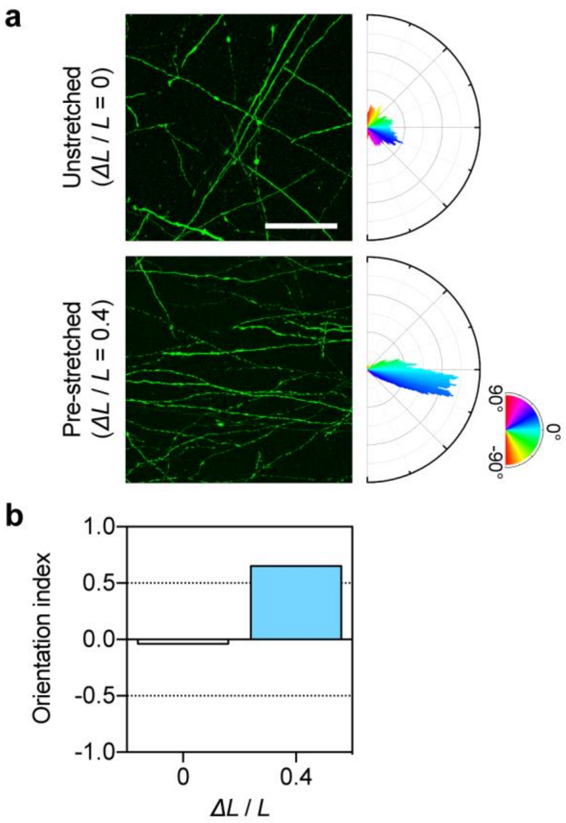

Supplementary Figure 5. Alignment of axons in anisotropically organized CA3-CA1 culture. a, Maximum intensity projection images (*left*) and polar frequency histograms of the orientation angles of axons (*right*) at DIV 21 in unstretched ($\Delta L/L$=0; *top*) and pre-stretched ($\Delta L/L$=0.4; *bottom*) PDMS chips (two right panels in Fig. 4e). Semicircular color index for the polar frequency histograms is presented in the bottom right corner. Scale bar, 50 μm. **b,** Orientation index of axons in unstretched ($\Delta L/L$=0) and pre-stretched ($\Delta L/L$=0.4) PDMS chips.

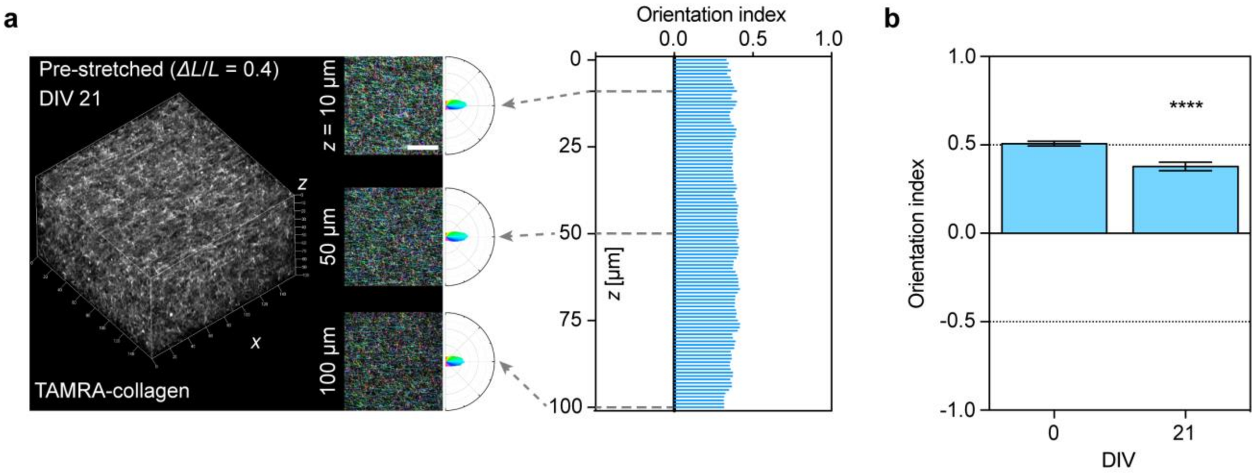

Supplementary Figure 6. Alignment of collagen fibers in cell-seeded, pre-stretched PDMS chips. a, Confocal fluorescence micrographs (*z*-stack) of TAMRA-labelled collagen fibrils rendered in 3D (160 μm × 160 μm × 100 μm) in a pre-stretched ($\Delta L/L$=0.4), cell-seeded PDMS chip. Insets on right show color-mapped confocal micrographs of collagen fibrils at depths of 10 (*top*), 50 (*middle*), and 100 (*bottom*) μm, as indicated. Polar frequency histograms of orientation angles are presented next to the three micrographs. Angular color scales for images and polar frequency histograms are identical to Fig. 1b. Scale bar, 50 μm. Graph on right shows orientation index of collagen fibrils throughout the depth of 100 μm. Gray arrows indicate the *z* position from which orientation indices were calculated. **b**, Orientation index of collagen fibrils averaged throughout the depth of 100 μm at DIV 0 and DIV 21. Error bars indicate standard deviation (100 focal planes). ****, $p<3.95\times10^{-95}$ (unpaired *t*-test).

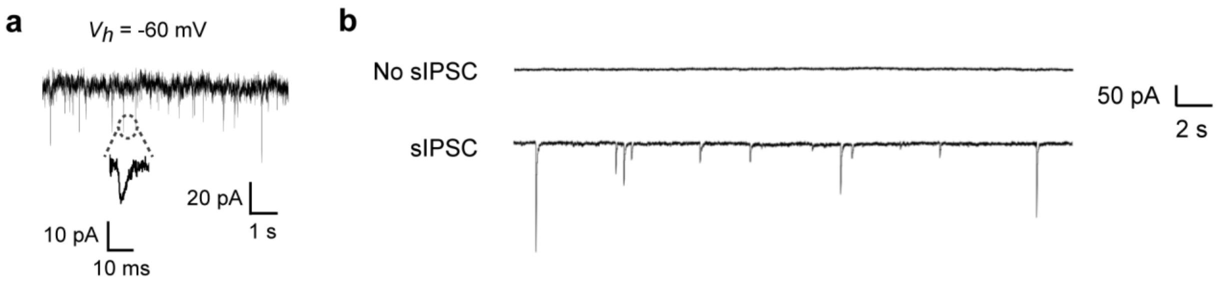

Supplementary Figure 7. Measurement of spontaneous postsynaptic currents recorded from CA1 neurons. Representative spontaneous excitatory postsynaptic currents (sEPSCs) (**a**) and inhibitory postsynaptic currents (sIPSCs) (**b**) from CA1 neurons. *Top trace* in **b**, a trace obtained from a CA1 neuron with no sIPSC. *Bottom trace* in **b**, a trace obtained from a CA1 neuron showing sIPSCs.

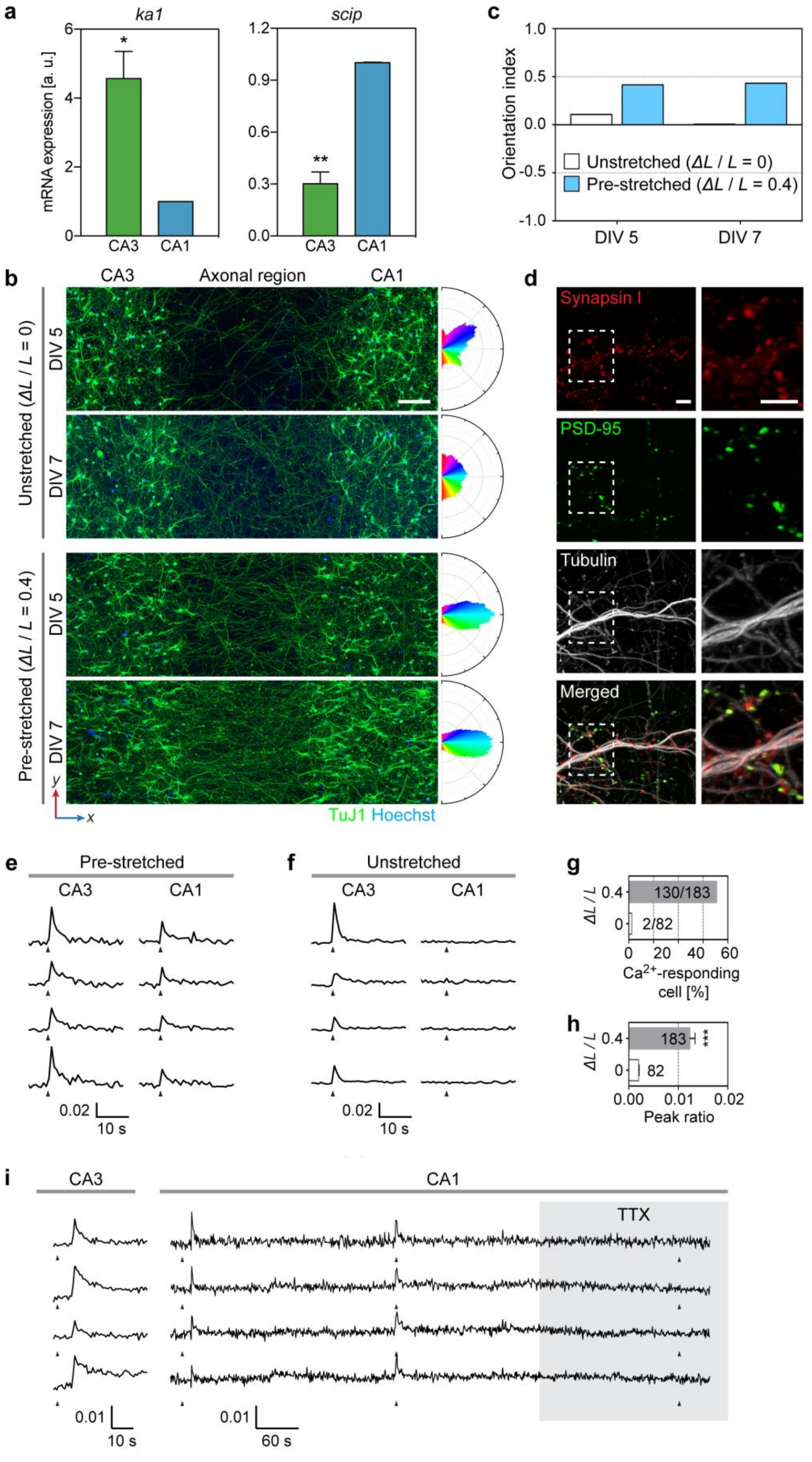
a
ka1
scip
mRNA expression [a. u.]
CA3
CA1
c
Orientation index
Unstretched (ΔL / L = 0)
Pre-stretched (ΔL / L = 0.4)
DIV 5
DIV 7
b
CA3
Axonal region
CA1
Unstretched (ΔL / L = 0)
Pre-stretched (ΔL / L = 0.4)
DIV 5
DIV 7
TuJ1 Hoechst
d
Synapsin I
PSD-95
Tubulin
Merged
e
Pre-stretched
f
Unstretched
0.02
10 s
g
ΔL / L
130/183
2/82
Ca2+-responding cell [%]
h
183
82
Peak ratio
i
CA3
CA1
TTX
0.01
10 s
60 s

1

Supplementary Figure 8. Formation of an anisotropically organized CA3-CA1 neural circuit with mouse neurons. a, Relative expression levels of *ka1* (*left*) and *scip* (*right*) mRNA in mouse primary hippocampal neurons isolated from areas CA3 and CA1. Levels are normalized to *gapdh* mRNA and presented in arbitrary units (a.u.). Error bars indicate standard error of mean (SEM) (n=3). * and **, p=4.50×10^{-2} (*ka1*) and 9.20×10^{-3} (*scip*), calculated from unpaired t-tests. **b** and **c**, Representative confocal fluorescence micrographs of CA3-CA1 cultures in unstretched ($\Delta L/L$=0) and pre-stretched ($\Delta L/L$=0.4) PDMS chips (**b**). Cultures were immunostained for neurites (TuJ1) at DIV 5 and 7, as indicated. *Right*, Polar frequency histograms of the orientation angles of neurites in the axonal region. Angular color scales are identical to Fig. 1**b.** Calculation of orientation indices of neurites in the axonal compartment is presented in **c**. **d,** Confocal fluorescence micrographs of the CA3-CA1 culture immunostained for synapsin I, PSD-95 and tubulin. Boxed areas are enlarged on right. Scale bars, 5 μm. **e-i,** A concentric bipolar electrode was placed in the CA3 region as in Fig. **7a**. **e** and **f,** Representative Ca^{2+} responses of fura-2/AM-loaded CA3 (*left*) and CA1 neurons (*right*), grown either in pre-stretched (**e**) or un-stretched (**f**) PDMS chips. **g,** Percentage of Ca^{2+}-responding cells in the CA1 region growing either in pre-stretched ($\Delta L/L$=0.4) or unstretched ($\Delta L/L$=0) PDMS chips. Numbers of total and responding cells are indicated. **h,** Average peak ratio of Ca^{2+} transients of CA1 neurons in pre-stretched ($\Delta L/L$=0.4) or unstretched ($\Delta L/L$=0) PDMS chips. Number of cells pooled from at least three independent recording sessions is indicated. Error bars indicate SEM. ***, p=5.833×10^{-11} (t-test). **i,** Representative Ca^{2+} responses evoked by repetitive electrical stimulation of CA3 neurons grown in pre-stretched ($\Delta L/L$=0.4) PDMS chips. Traces show Ca^{2+} responses in the absence or presence of TTX, as indicated. **e**, **f**, and **i**, Triangles indicate electrical stimulation (1 s, 20 Hz).

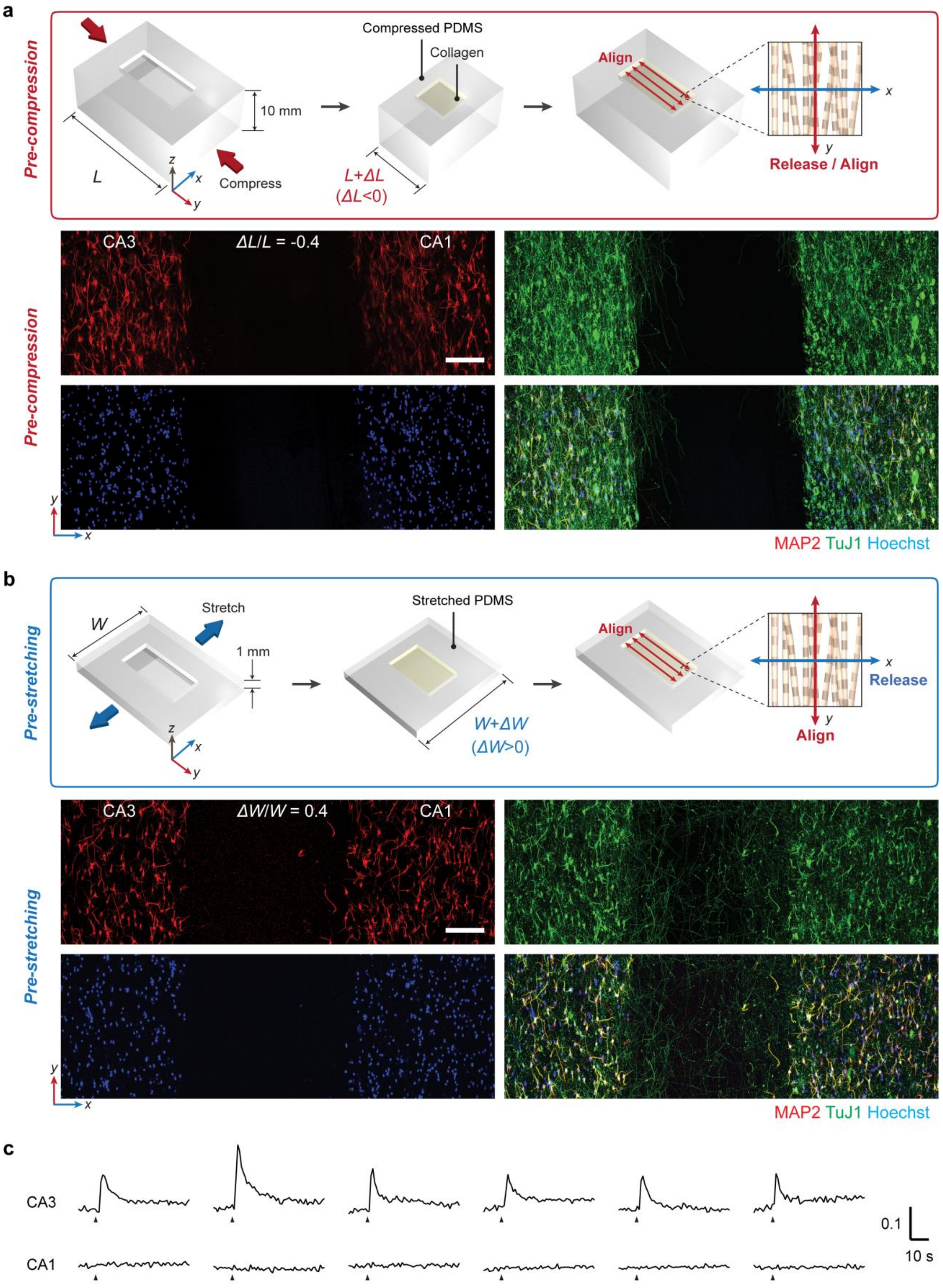
a
Pre-compression
Compressed PDMS
Collagen
Align
10 mm
L
Compress
L+ΔL
(ΔL<0)
x
y
z
Release / Align
CA3
ΔL/L = -0.4
CA1
MAP2 TuJ1 Hoechst
b
Pre-stretching
W
Stretch
1 mm
Stretched PDMS
W+ΔW
(ΔW>0)
Release
Align
CA3
ΔW/W = 0.4
CA1
MAP2 TuJ1 Hoechst
c
CA3
CA1
0.1
10 s

1

Supplementary Figure 9. Alignment of collagen fibrils along *y*-axis fails to support CA3-CA1 connectivity. **a**, *Top*, Schematic for inducing alignment along *y*-axis by pre-compression of 10 mm-thick PDMS chip along the *y*-axis. *Bottom*, representative confocal fluorescence micrographs of the CA3-CA1 culture (DIV 7) showing dendrites (MAP2; red), axons (TuJ1; green) and nuclei (Hoechst; blue). Scale bar, 200 μm. **b**, *Top*, Schematic for inducing alignment along *y*-axis by pre-stretching 1 mm-thick PDMS chip along the *x*-axis. *Bottom*, representative confocal fluorescence micrographs of the CA3-CA1 culture (DIV 7) showing dendrites (MAP2; red), axons (TuJ1; green) and nuclei (Hoechst; blue). Scale bar, 200 μm. **c**, Representative Ca^{2+} responses of fura-2/AM-loaded CA3 and CA1 neurons at DIV 22, grown PDMS chips aligned along *y*-axis.

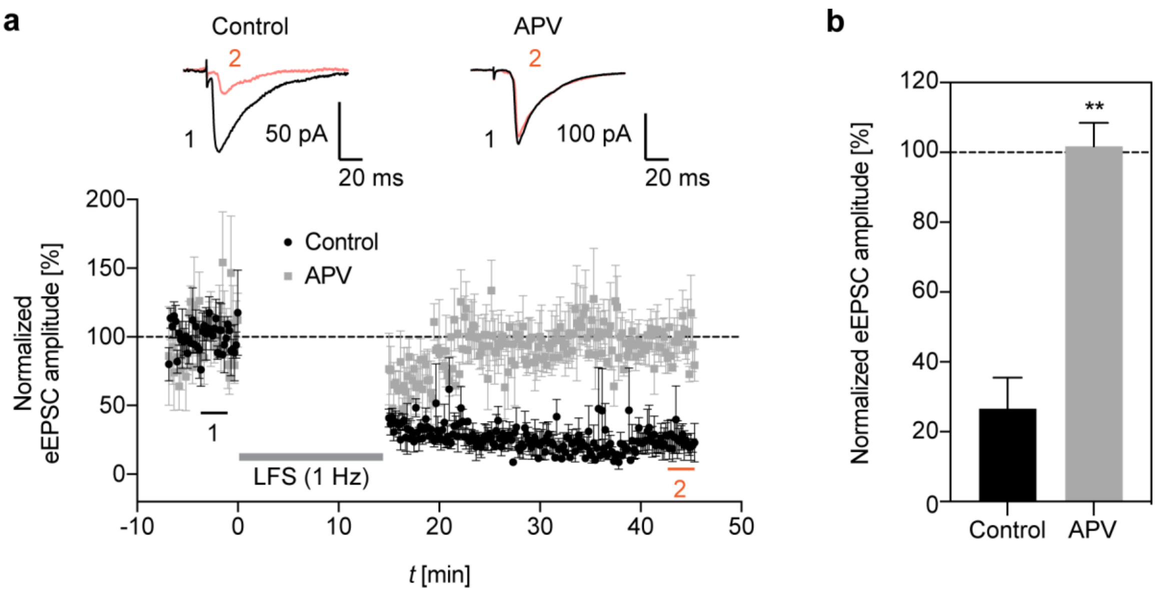

Supplementary Figure 10. Measurement of long-term depression (LTD) in anisotropically organized CA3-CA1 culture. a, Graphs represent evoked EPSC (eEPSC) amplitude recorded from CA1 neurons with control solution (black) or APV-containing solution (gray) in each batch of anisotropically organized 3D culture. The LTD was induced by stimulation at 1 Hz for 15 min in the CA3 area. Inset traces represent eEPSCs measured from one neuron during baseline (1) and at the indicated time after low frequency stimulation (2). Error bars indicate standard error of mean (n=3 for each). **b,** Relative eEPSCs analyzed at 45 min from **a**. **, $p=2.56\times10^{-3}$ (t-test).

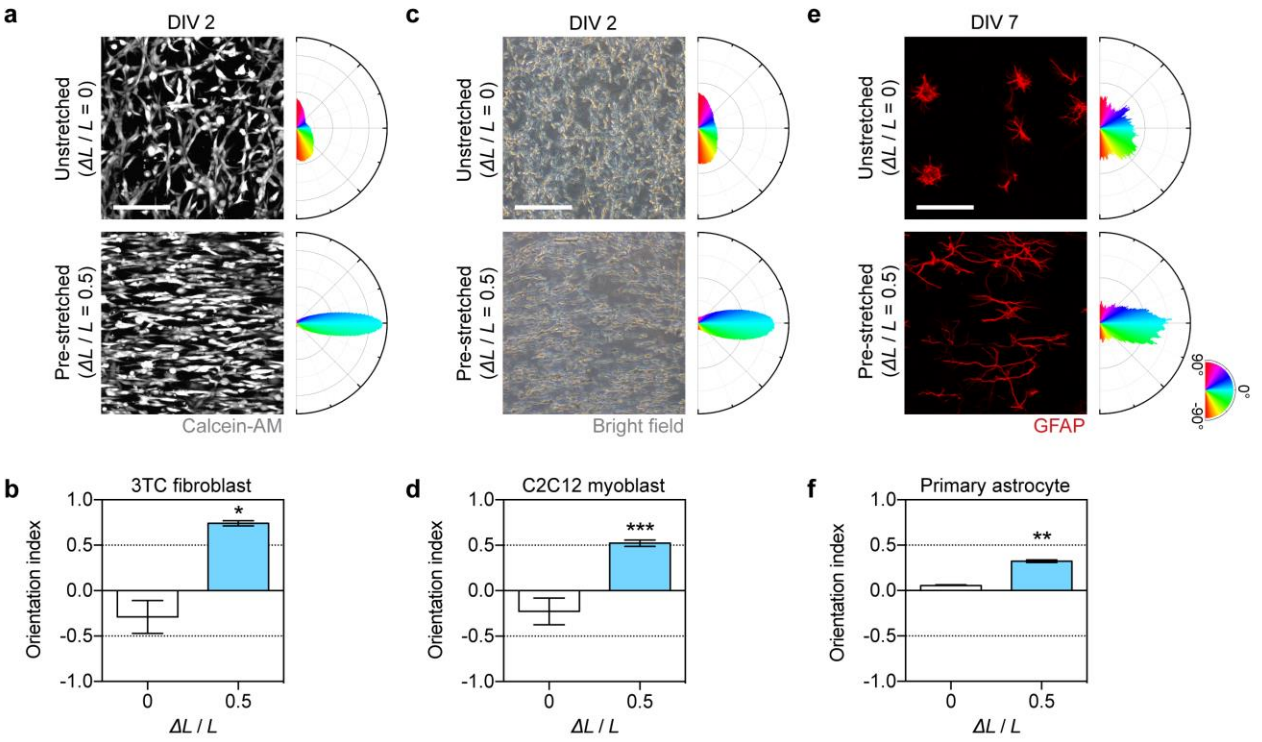

Supplementary Figure 11. Alignment of 3T3 fibroblast, C2C12 myoblasts, and primary astrocytes induced by mechanical deformation of PDMS chips. a, c, and **e**, Images of 3T3 fibroblasts (**a,** calcein-AM-loaded images; DIV 2), C2C12 myoblasts (**c,** bright field images; DIV 2), and primary astrocytes (**e,** GFAP-stained images; DIV 7) growing in unstretched (*top*) and pre-stretched (*bottom*) PDMS chips. Cell-embedded collagen solutions were loaded into single-channel PDMS chips, either unstretched ($\Delta L/L$=0, t_D=5 min) or pre-stretched ($\Delta L/L$=0.5, t_D=5 min), following the processes illustrated in Fig. 1a. Polar frequency histograms of the orientation angles are shown on right. Semicircular color index for the polar frequency histograms is presented in the bottom right corner. Scale bar, 50 (**a** and **c**) and 100 (**e**) μm. **b, d, and f,** Orientation indices of 3T3 fibroblasts (**b**), C2C12 myoblasts (**d**), and primary astrocytes (**f**) growing in unstretched ($\Delta L/L$=0) and pre-stretched ($\Delta L/L$=0.5) PDMS chips. Error bars indicate standard deviation. *, ***, and ** denote statistical differences with p values of 1.54×10^{-2}, 1.0×10^{-3}, 1.9×10^{-3}, respectively (t-tests).
